# Alpha event-related decreases during encoding in adults with ADHD – An investigation of sustained attention and working memory processes

**DOI:** 10.1016/j.bbr.2024.115003

**Published:** 2024-04-19

**Authors:** René Freichel, Nicolas Zink, Fang Yu Chang, Juan Diego Vera, Holly Truong, Giorgia Michelini, Sandra K. Loo, Agatha Lenartowicz

**Affiliations:** a Department of Psychology, University of Amsterdam, The Netherlands; b Semel Institute for Neuroscience and Human Behavior, University of California, Los Angeles, United States; c Department of Psychology, University of California Los Angeles, Los Angeles, United States; d Department of Psychiatry and Biobehavioral Sciences, University of California, Los Angeles, United States; e Department of Biological & Experimental Psychology, School of Biological and Behavioural Sciences, Queen Mary University of London, London, UK

## Abstract

**Background:**

Executive functioning deficits are central to established neuropsychological models of ADHD. Oscillatory activity, particularly the alpha rhythm (8–12 Hz) has been associated with cognitive impairments in ADHD. However, most studies to date examined such neural mechanisms underlying executive dysfunction in children and adolescents with ADHD, raising the question of whether and to what extent those ADHD-related working memory impairments are still present in adults. To this end, the current study aimed to investigate the role of alpha event-related decreases (ERD) during working memory processes in adults with and without ADHD.

**Methods:**

We collected electroencephalographic (EEG) data from 85 adults with a lifetime diagnosis of ADHD and 105 controls (aged 32–64), while they performed a continuous performance (CPT) and a spatial delayed response working memory task (SDRT). Time-frequency and independent component analysis (ICA) was used to identify alpha (8–12 Hz) clusters to examine group and condition effects during the temporal profile of sustained attention and working memory processes (encoding, maintenance, retrieval), loads (low and high) and trial type (go and nogo).

**Results:**

Individuals with ADHD exhibited higher reaction time-variability in SDRT, and slower response times in SDRT and CPT, despite no differences in task accuracy. Although working memory load was associated with stronger alpha ERD in both tasks and both groups (ADHD, controls), we found no consistent evidence for attenuated alpha ERD in adults with ADHD, failing to replicate effects reported in children. In contrast, when looking at the whole sample, the correlations of alpha power during encoding with inattention and hyperactivity-impulsivity symptoms were significant, replicating prior findings in children with ADHD, but suggesting an alternate source for these effects in adults.

**Conclusions:**

Our results corroborate the robustness of alpha as a marker of visual attention and suggest that occipital alpha ERD normalizes in adulthood, but with unique contributions of centro-occipital alpha ERD, suggesting a secondary source. This implies that deviations in processes other than previously reported visuospatial cortex engagement may account for the persistent symptoms and cognitive deficits in adults with a history of ADHD.

## 1. Introduction

Attention-deficit/hyperactivity disorder (ADHD) is a neurodevelopmental condition marked by symptoms of inattention, hyperactivity, and impulsivity that affect around 2–7% of children and adults globally [45,48]. Historically, ADHD has been predominantly perceived as a childhood disorder due to its onset in childhood with diminishing symptoms over the course of adolescence [8]. However, longitudinal studies in the last decade showed evidence for both persisting inattentive and hyperactive/impulsive ADHD symptomatology [23,47] and an emerging group of adult ADHD cases with no record of childhood-onset [43]. Thus, there is a need to understand the mechanisms of impairments present in adults with ADHD.

ADHD is associated with a range of cognitive and neural alterations reported in children, adolescents, and adults [11]. These include changes in executive functions, such as working memory, inhibition, and attentional control [38] that are closely related to symptoms of hyperactivity-impulsivity and inattention. Some evidence suggests that impairments in these various executive functions, such as working memory, inhibition, and vigilance are associated with symptom severity in ADHD [34,44,5]. Several neurocognitive models have been proposed to integrate ADHD-specific alterations in both sensorimotor visual processes as well as higher-level executive functioning [2,46,49]. For instance, one prominent model posits that weak inhibitory control may stem from alterations in the frontodorsal striatal circuit and associated processes [49].

Brain oscillations measured through electroencephalography (EEG) have emerged as putative neurocognitive markers of executive functioning [4,54] and have been recently associated with group effects, symptoms and outcomes in ADHD [26,41,51]. Neural modulation of oscillations in the alpha frequency band (8–12 Hz) has been proposed as a key marker of attentional shifting and engagement [19] associated with performance in executive functioning in a broad dorsal attention network [55]. In ADHD, alpha power modulation during stimulus processing in a working memory task was shown to be a biomarker of attentional impairment in predominantly children with ADHD [27,41]. Existing research points to an attenuated event-related decrease (ERD) of alpha-band activity during the encoding stages of a working memory task [24]. A concurrent EEG-fMRI study in pre-adolescents with ADHD, found that the attenuated alpha ERD during stimulus encoding was associated with weakened occipital activation [25]. These attenuated alpha ERD effects appear to be primarily present during visual selective attention, in particular in the ADHD inattentive type [27]. Modulation in the alpha band has also been linked with clinically relevant outcomes beyond its role as a marker of attentional impairment. Alpha modulation during encoding was associated with poorer executive functioning and elevated ADHD symptoms [29]. Moreover, measures of rightward alpha asymmetry [17] and alpha ERD occipital connectivity [25] were shown to be associated with a greater number of ADHD symptoms.

However, most studies on attenuated alpha ERD effects focused on children and adolescents and less is known about altered oscillatory alpha activity in adults with ADHD. Furthermore, the existing studies of alpha ERD effects in adults with ADHD mostly rely on small to moderate sample sizes (n = 69, [6]; n = 25, [12]; n = 21, [18]; n = 136, [30]; n = 20, [40]; n = 15, [42]). Existing studies of alpha ERD among children and adolescents examined alpha at both the source (i.e., based on independent component analysis) [24,29] and the channel level [1,39]. Similarly, the relationship between alpha ERD and different behavioral and clinical outcomes in adult samples with ADHD remain poorly understood. The goal of our study was to investigate the role of alpha ERD during different working memory processes in adults with ADHD. We extend existing studies on alpha ERD in two meaningful ways: (1) our sample consists of a large number of adult participants with a lifetime diagnosis of ADHD and controls with a wide age spectrum; (2) by including tasks of both sustained attention (Continuous Performance Task) and working memory (spatial delayed response task), we are able to study the role of alpha ERD and disentangle its relationship to behavioral performance (i.e., response speed and accuracy) in contexts with different cognitive demands. In doing so we test whether associations found between alpha modulation and ADHD symptoms in children would also replicate in adults. Based on existing research conducted on children with ADHD [29], we predicted that 1) adults would show attenuated alpha ERD effects during working memory encoding, and 2) alpha ERD would correlate with both hyperactivity-impulsivity and inattention symptoms.

## 2. Material and methods

### 2.1. Participants

The sample consisted of 190 participants who participated in a family study on the genetics of ADHD. Only families with at least one child with ADHD were eligible to take part in the study. Individuals were excluded from the study if they had a history of neurological disorders, had suffered a head injury resulting in concussion, had a lifetime diagnosis of schizophrenia or autism, or an Intelligence Quotient (IQ) less than 70. Participants in the ADHD group (*n* = 85) all received a lifetime (past or present) diagnosis of ADHD. Participants in the control group (*n* = 105) were individuals that did not receive a lifetime diagnosis of ADHD and did not fulfill the criteria for a current diagnosis. All participants in this community sample were recruited through local advertisements, schools, and organizations. After providing informed consent, all participants were invited to an in-person lab study visit. The study design and sample selection are described in more detail elsewhere [31,32,33]. The study received ethical approval by the UCLA Institutional Review Board.

### 2.2. Procedure

During the study visit, all participants completed clinical interviews and a battery of cognitive tasks during which EEG recordings took place. Participants were interviewed using the Schedule for Affective Disorders and Schizophrenia (SADS-LAR, [37] and additional Behavioral Disorders supplements, including the Schedule for Affective Disorders and Schizophrenia for School-Age Children (KSADS-PL, [20]). These interviews were used to establish a ‘best estimate’ diagnosis of lifetime ADHD (past and/or current). All clinical interviews were conducted by trained interviewers with extensive experience. Further details on the procedures and interview can be found in [33]. Individuals with a lifetime diagnosis of ADHD were classified into the ADHD group. Participants who did not receive a lifetime diagnosis of ADHD were classified in the control group.

### 2.3. Task measures

#### 2.3.1. Continuous performance task

The Continuous Performance Test (CPT) [9] is a widely used measure of response inhibition and sustained attention. Participants viewed letter stimuli and were instructed to press and release the space bar as quickly as possible after every letter (*A, B, C, D, F, I, L, O*, and *T*), except after the appearance of the target letter (*X*). The computerized task lasted 14.5 minutes and included 360 trials with 36 targets that were presented for 250 ms in a random order. Three different inter-stimulus-interval (ISI) times (1000, 2000, 4000) were varied across trials to capture individuals’ level of vigilance. We examined block-wise reaction times and accuracies for correct trials.

#### 2.3.2. Spatial delayed response task

The Spatial Delayed Response Task (SDRT) [16,50] is a commonly used behavioral measure of spatial working memory. In each trial, participants were shown 1, 3, 5 or 7 yellow dots on a black screen (target stimulus) and instructed to encode their location. Following a maintenance interval during which the screen showed only a fixation cross, a single green dot (probe stimulus) was shown. Participants were instructed to decide whether the probe stimulus matches the location of any of the yellow dots shown during encoding. The location of the probe stimulus matched one location of the target stimulus in 50% of the trials. Every trial started with a 500 ms fixation cross, was followed by the target stimulus presented for 2000 ms (encoding phase), then a 3000 ms black screen with a fixation cross followed (maintenance phase) and finally, in the retrieval phase, the probe stimulus was presented for 3000 ms and participants were instructed to decide and respond whether the probe matches any of the target stimulus locations. Participants were instructed to press the left arrow or right arrow buttons to indicate their choice (same location “match” or different location “non-match”). The number of simultaneously presented dots (1, 3, 5, or 7) was varied during encoding, randomized across trials, to manipulate memory load. The inter-trial period, during which the screen was blank, was 2000 ms. There were 24 training trials and subjects were required to achieve at least 50% accuracy to be able to start the experiment blocks. In total, there were 2 blocks with 48 trials in each. The SDRT lasted 17 minutes in total. Dependent variables included the block-wise reaction times and accuracies for correct trials.

### 2.4. EEG recording & preprocessing

During both SDRT and CPT tasks, EEG recordings were collected. EEG signals were acquired using a 40 Ag/AgCl surface electrode cap in a 10/20 location system, sampled at 256 Hz. and referenced to linked ears. Electrode locations on the scalp were recorded for each individual using Fowler calipers.

The EEG data were processed using custom MATLAB (*The Math-Works, Natick, MA*) scripts based on the EEGLAB toolbox [13]. We followed standard preprocessing protocols similar to the procedures described in Lenartowicz et al. [29] and Lenartowicz et al. [24]. EEG data were high pass filtered (> 1 Hz) and manually inspected for noisy signal. We applied independent component analysis (ICA) [35] to single-subject EEG data for both data cleaning and to identify ICs of interest for subsequent analysis. For data cleaning, ICs corresponding to eye blinks, movement or muscle artifacts were manually identified and disregarded in all further analysis steps. The remaining ICs were retained for clustering and group analyses as described below.

Following preprocessing, the data were segmented into epochs. The epochs were time-locked to the onset of the encoding stimulus and included the entire trial duration. EEG power was obtained on IC time courses using Morlet wavelets, extracted in 1 Hz bins, and log-transformed to decibel (dB) units. The ICs of all subjects were then clustered using EEGLAB’s PCA-based k-means clustering based on similarity in space, time and frequency dimensions, to identify clusters of ICs that are both common across all participants and capture independent sources of signal. We selected relevant clusters for further analysis based on both the topography and time-series of the IC clusters, as described in Lenartowicz et al. [24,29]. In the current analysis we focused on clusters with occipital, central, and frontal topography, as these capture the effects of interest based on our priors as described below. This investigation of effects at the level of the IC source space was considered appropriate as it 1) provides better signal-to-noise ratio (compared with single channels, [36]) and 2) reduces dimensionality for all statistical comparisons (i.e., fewer multiple comparisons). We provide reference channel effects in the supplementary materials Section 6.

Features of interest, namely event-related spectral perturbations (ERSPs), for group analyses were then extracted from the IC time courses for each subject in the selected clusters. We focused our analyses on ERSPs in the alpha range (8–12 Hz). We computed mean event-related spectral perturbations (ERSPs) for each task following stimulus onset by dividing the frequency power by that obtained during the baseline (i.e., − 1000 to − 600 ms for SDRT, − 900 to –300 ms for CPT) and log-transforming the result to decibel units (dB). We averaged frequencies between 8 and 12 Hz. These signals were obtained for each individual in each task and each cluster of interest and used in all group analyses. For each task, we only report results for central-occipital and occipital alpha clusters in the manuscript. Additional clusters (frontal and central for SDRT) were not significant and are provided in the supplementary materials.

### 2.5. Clinical measures

The ADHD Rating Scale (ADHD-R) [15] is a widely used measure of ADHD symptom severity. The rating scale includes 18 indicators (9 hyperactivity-impulsivity, 9 inattention) that are rated on a scale from 0 (never or rarely) to 3 (very often). An example indicator is “Fail to give close attention to details or makes careless mistakes in my work.”.

### 2.6. Data analysis

All statistical analyses were conducted in RStudio (R [10]). For the analysis of the SDRT task, we used separate ANOVAs to test (diagnostic) group, condition (high vs low load), and group × condition effects in each of 500 ms windows across the entire duration of a-priori specified encoding phase. For the analysis of the CPT task, we used the same approach with condition including go versus no-go trials instead of load. For both tasks, we also tested group × condition effects throughout the entire trial. To do so, we averaged 500 ms windows of alpha power (8–12 Hz averages) and used ANOVAs to test group and condition effects at all time windows. In all analyses, we also modelled group × load/condition. To control the overall Type I error rate when performing multiple exploratory ANOVA tests, we used the false discovery rate (FDR) method to adjust the p-values for each effect (load/condition, group, load/condition × group) separately. We report corrected p-values in the results section.

To examine associations between alpha activity and task/symptom measures, we used linear regression models that included gender at birth (female/male) as a predictor. We first ran a principal component analysis (PCA) to extract components for both tasks that represent accuracy, reaction-times, and reaction-time variability respectively. The PCA was used to reduce multiple behavioral measures (i.e., task-based reaction-time/accuracy measures) to a lower dimension. Further information on selecting principal components can be found in the supplementary materials Section 1. We obtained two components for the CPT task representing accuracy and reaction-time respectively. For the SDRT task, we obtained three components representing accuracy, reaction-time and reaction-time variability respectively. The extracted components were used in analyses of associations between behavior and alpha power across individuals.

## 3. Results

### 3.1. Sample characteristics

The sample demographics are presented in Table 1. The ADHD and control groups did not significantly differ with respect to age or sex. Individuals with a lifetime diagnosis of ADHD reported more symptoms on both the inattention and hyperactivity-impulsivity subscales, compared with the control group. When examining performance measures on the SDRT and CPT tasks, individuals in the ADHD group were slower to respond and had higher reaction-time variability at lower loads compared to controls (see Table 1). Moreover, individuals in the ADHD group were slower to respond (in CPT block 2) compared with the control group.

### 3.2. Spatial working-memory (SDRT)

#### 3.2.1. Central occipital cluster

**Fig. 1.**
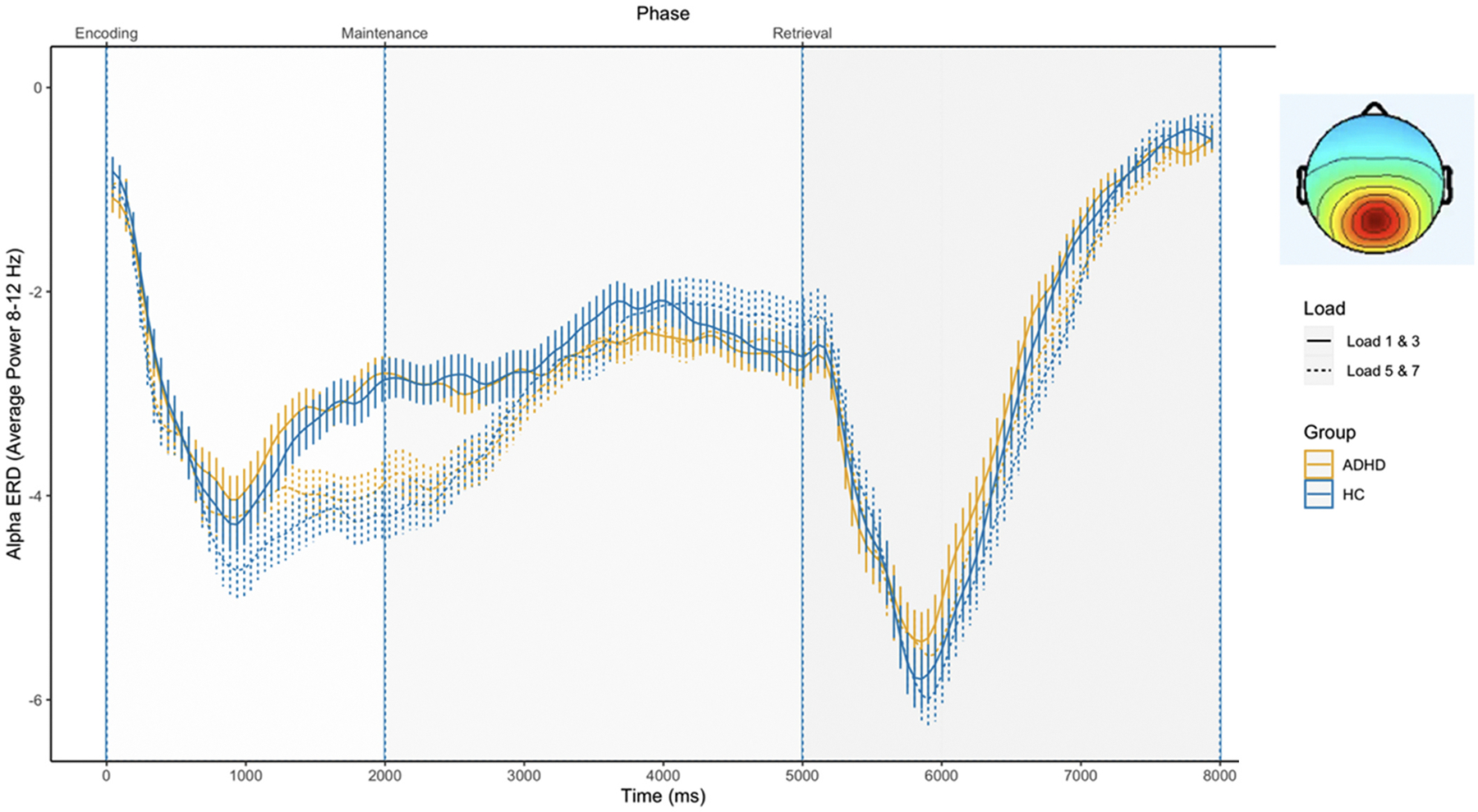
Alpha power in the Central-occipital cluster during the SDRT task. *Note*. The stimulus onset occurs at time = 0. Standard errors are shown in vertical bars. HC = control group.

Fig. 1 shows the averaged alpha power time course during the SDRT trials at the central-occipital cluster. During the encoding phase (0–2000 ms), there was a significant drop in alpha power (i.e., the event-related decrease or ERD). Significant differences between loads (load effects, *p* < 0.05, time windows: 500–1000 ms, 1000–1500 ms, 1500–2000 ms, 2000–2500 ms, 2500–3000 ms, 6500–7000 ms; *F* = 5.969–100.292) emerged late in encoding and into the maintenance phase, with stronger alpha ERD for higher working memory loads (loads 5 & 7) compared to lower loads (load 1 & 3). We found no significant group differences in alpha power, at any time interval (see Table S5, *p* > 0.05, *F* = 0.002–1.325).

#### 3.2.2. Occipital cluster

**Fig. 2.**
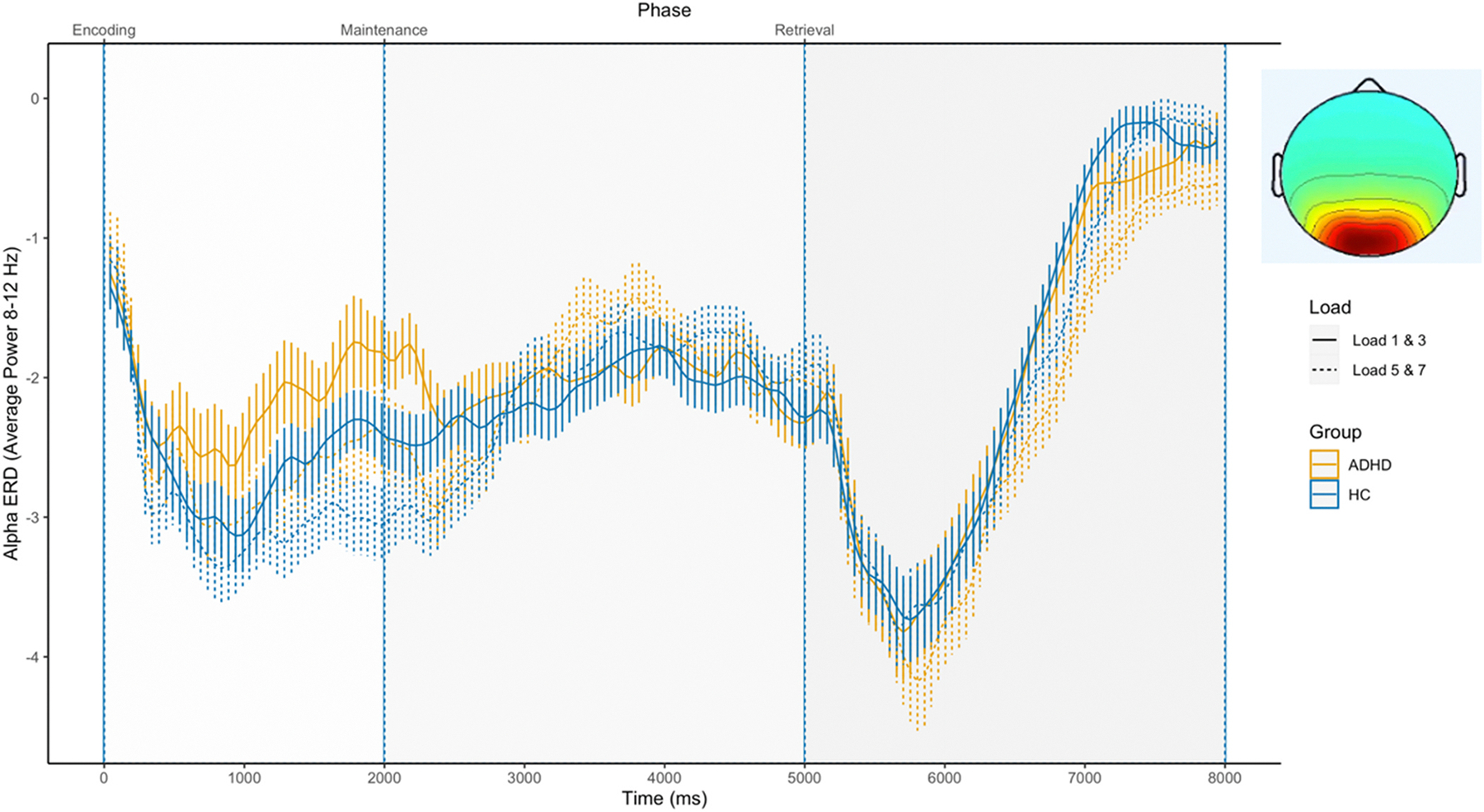
Alpha power in the Occipital cluster during the SDRT task.

The alpha power time course for the occipital cluster is shown in Fig. 2 below. There were significant effects of working memory load during stimulus processing periods with higher alpha ERD at higher loads (time windows: 500–1000 ms, 1000–1500 ms, 1500–2000 ms, 2000–2500 ms, 6500–7000 ms, 7000–7500 ms; F = 7.559–35.493, see Table S7). We found no significant group differences in alpha between ADHD and control groups at the occipital cluster (p > 0.05, F = 0.003–4.599, see Table S7).

#### 3.2.3. Alpha modulation during spatial working memory and its association with behavior and symptoms

**Fig. 3.**
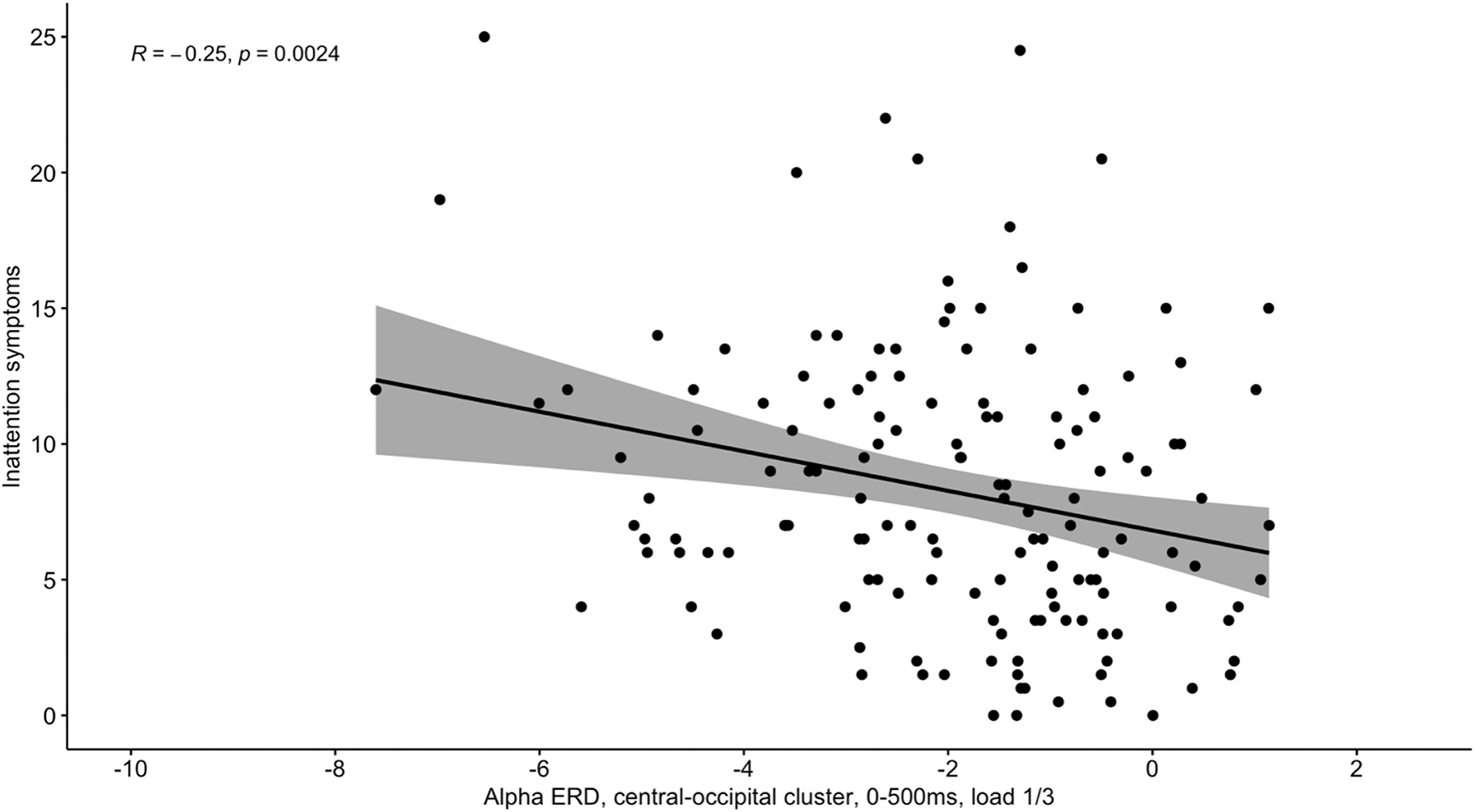
Associations between symptoms and alpha occipital alpha power ERD.

We examined associations between alpha power ERD and both behavior and clinical symptoms for the central-occipital and occipital clusters. Central-occipital alpha power during lower loads was significantly associated with inattention symptoms (*r*=−0.25, p<0.01, see Fig. 3) and showed trend-level significant associations (*r*=−0.15, *p*=0.07) with hyperactivity-impulsivity symptoms (see Table S9). When repeating the analysis only in the ADHD group, the associations become insignificant, yet show a similar direction as the effects found for inattention in the entire sample (*r* = −0.16, *p* = 0.19). Thus, it is possible that these associations are driven by group differences. Weaker encoding central-occipital alpha power ERD during lower loads was associated with fewer symptoms. In other words, a stronger alpha ERD at the central-occipital cluster was associated with more inattentive participants

We found no significant associations between occipital alpha power and symptom measures (see Tables S11). We also examined associations between alpha power and behavioral task measures (see Tables S12). Interestingly, we found that encoding central-occipital alpha power was significantly associated with task accuracy at higher loads (see Table S10). Weaker central-occipital alpha ERD was associated with higher task accuracy, which is complementary to its association with symptoms. There were no significant associations between alpha at other clusters and performance measures (see supplement).

### 3.3. Sustained attention (CPT task)

#### 3.3.1. Central-occipital cluster

**Fig. 4.**
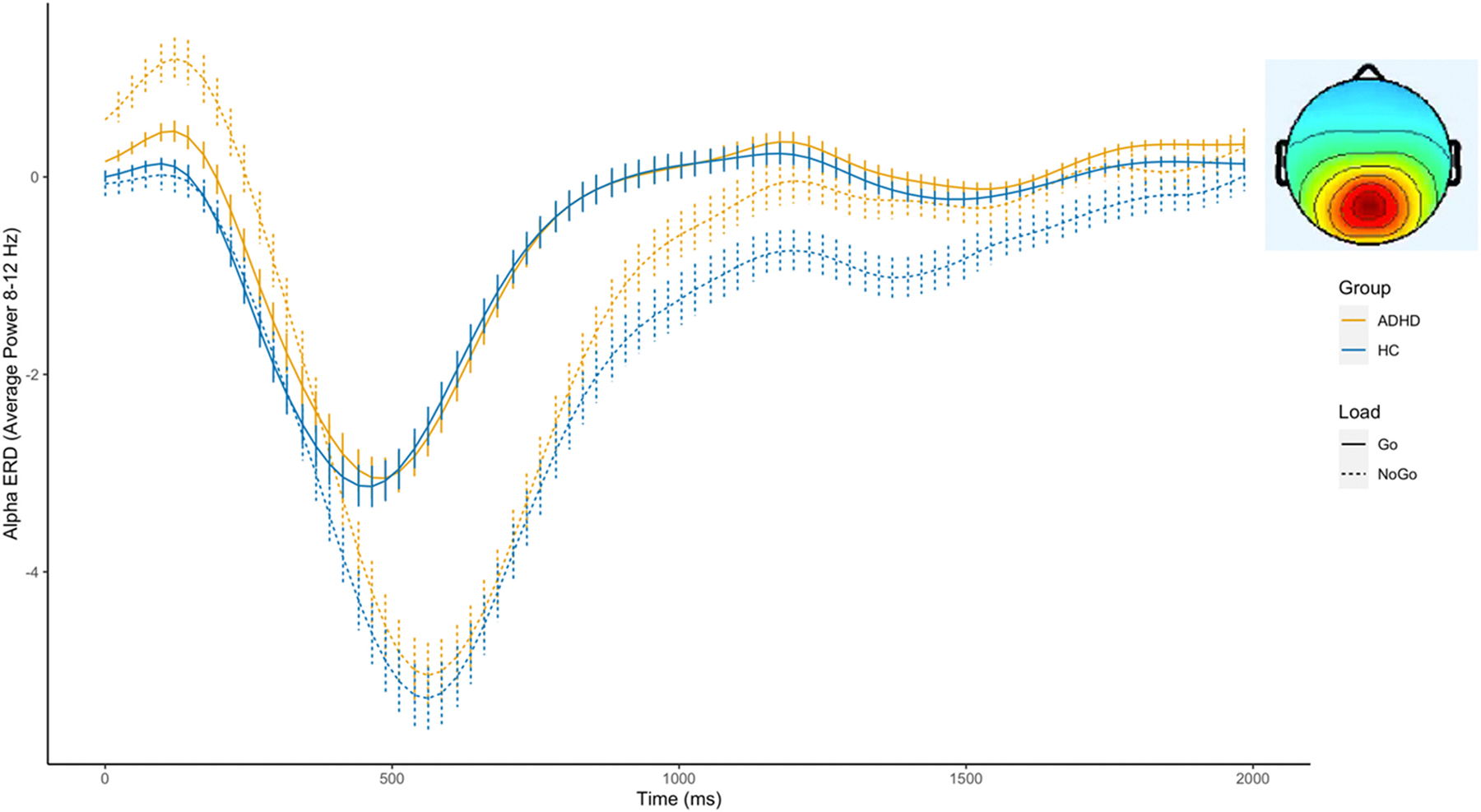
Alpha power in the Central-occipital cluster during the CPT task.

Fig. 4 shows the alpha power time course during the CPT task. There was a substantial drop in alpha power after stimulus onset. We found significant differences between conditions with stronger alpha ERD during the NoGo compared to the Go conditions, particularly within the 500–1000 ms (F = 238.795, p <0.01) and 1000–1500 ms (F = 27.917, p < 0.01) time windows. Group differences were significant in the 0–500 ms window (F = 6.894, p < 0.01), and a significant Group × Condition interaction was found in the same window (F = 7.244, p < 0.01), indicating attenuated alpha ERD in the ADHD group compared to the control group, especially in the NoGo condition. This interaction effect persisted into the 1000–1500 ms window (F = 5.372, p < 0.05). Group differences, signifying attenuated alpha power in the ADHD group compared with the control group, were again significant in the 1500–2000 ms window (F = 4.224, p < 0.05). We found no significant associations between central-occipital alpha power and symptoms or behavioral measures (see Tables S17–S18).

#### 3.3.2. Occipital cluster

The occipital alpha power cluster shows similar condition effects with stronger alpha ERD in the NoGo compared with the Go condition (time windows: 500–1000 ms, 1000–1500 ms, 1500–2000 ms; F = 4.561–45.85, see Table S13). We found no significant group differences or group × condition interactions (p > 0.05, F = 0.001–1.078, Table S13).

#### 3.3.3. Alpha modulation during sustained attention and its association with behavior and symptoms

There were no significant associations between occipital alpha power and inattention or hyperactivity-impulsivity symptoms (see Figure S15 in the supplementary materials). Also, we found no significant associations between alpha power clusters (central-occipital and occipital) and behavioral measures (RT and accuracy components); see Figures S16/S18 in the supplementary materials.

## 4. Discussion

The present study is one of the first to evaluate differences in alpha power modulation, and its association with behavioral and symptom measures among a large sample of adults with and without a lifetime diagnosis of ADHD. We found that adults with a lifetime diagnosis of ADHD exhibited elevated inattention and hyperactivity-impulsivity symptoms alongside a slower response time and greater reaction-time variability. However, against our predictions, occipital alpha power modulations did not significantly differ between the ADHD and control groups. Importantly, across the entire sample, though not within the ADHD subgroup specifically, our analysis revealed a significant association between centro-occipital alpha ERD attenuation and the severity of ADHD symptoms, particularly inattention, thereby replicating previously found links in children and adolescent samples. However, differences in the source location and direction of this association suggest deviant processes in the adult sample.

### 4.1. Persisting symptoms and cognitive impairments in adults with a lifetime diagnosis of ADHD

Individuals with a lifetime diagnosis of ADHD reported a significantly higher symptom burden, specifically more symptoms of inattention and hyperactivity-impulsivity compared to individuals without ADHD. This is in line with reports indicating that symptoms in both domains can persist over longer periods into adulthood [47]. Interestingly, we also found differences in behavioral performance in two gold-standard cognitive tasks, namely a sustained attention (continuous performance) task (CPT) and a spatial delayed-response working memory task (SDRT). Individuals in the lifetime ADHD group exhibited slower response times and higher reaction-time variability at lower working memory loads compared with individuals without ADHD. Interestingly, we found these significant group differences only at lower working memory loads. This could suggest that individuals with ADHD may use compensatory strategies (see [22]), such as an increased effort to mitigate cognitive difficulties in situations where working memory demands are elevated (higher loads). This finding is also consistent with a prior report showing that children with ADHD showed attenuated alpha ERD particularly when the task difficulty was low [24].

### 4.2. Alpha ERD as a signature of visuospatial processes

Our findings are, however, consistent with prominent interpretations of alpha ERD as related to cortical excitability of the visuospatial cortex, replicating its association with attentiveness. We show significant effects of working memory load on alpha ERD during the maintenance phase. Higher working memory loads elicited stronger alpha ERD compared to lower loads. This aligns with previous studies [21,29] and is consistent with the prior interpretation that alpha ERD may reflect the activation of visual information for enhanced working memory performance [53]. Similarly, we found significant condition effects with stronger alpha ERD during NoGo compared with Go trials. Alpha ERD in the present study is consistent with increased cortical engagement with increased demands for information processing. To validate this interpretation we ran additional supplemental analyses (Supplementary Material Section 6, see Figure S14, *p* <0.05), and replicated prior reports of alpha ERD being negatively associated with the P3 event-related potential [28], a robust marker of updating processes (see [14] and Polich, 2007). As such, our data is consistent with the interpretation of alpha ERD as an indicator of the degree of visual encoding processes.

### 4.3. Normalization of visuospatial processes in adults?

Importantly, contrary to our hypothesis, we found no significant group differences in alpha ERD between the ADHD and control groups in the central-occipital and occipital alpha clusters. This finding does not fit with the attenuated alpha ERD effects found during encoding in children with ADHD [24,3,7]. Our results might be interpreted as evidence in favor of the normalization of visuospatial processes (i.e., functional signature of alpha ERD) with development. However, the presence of significant performance differences, along with differences in symptoms, suggests a persisting deficit in adults with a lifetime diagnosis of ADHD that, perhaps, may be associated with processes other than visuospatial attention processes. This interpretation is consistent with the finding of alpha ERD correlations with symptoms and behavioral task performance at the central-occipital cluster in the entire sample rather than the inferior occipital cluster, as has been previously reported [29]. In addition, the correlations were in the opposite direction, whereby stronger alpha ERD was associated with worse performance and greater symptoms. The presence of this effect at lower loads complements the significant group differences in behavioral performance at lower loads. This phenomenology is consistent with a developmental deviation that while accompanied by apparently normalized visuospatial occipital responses, is also accompanied by a secondary source that is unique to individuals with higher inattention and lower task accuracy.

The interpretation of this source deserves further consideration and research. It could reflect the product of atypical childhood visual processing, potentially a compensatory process. This would explain why stronger alpha ERD here was associated with more symptoms. Such an interpretation could be tested in longitudinal studies. The effect could also reflect an anomaly in a subgroup of adults in our sample. For instance, a recent excitation/inhibition model suggests that adults with ADHD may show attenuated or elevated alpha power depending on individuals’ biotype (trait-like subgroup) [12]. Thus, future work should examine alpha ERD and its association with clinical and behavioral outcomes for each individual’s biotype independently. Moreover, arousal-related theories of ADHD [52] suggest that arousal dysregulation during task performance may account for inattention and hyperactivity-impulsivity symptoms. Teasing apart these alternate putative causal pathways will be an important future direction of this work.

### 4.4. Temporal dynamics of alpha ERD

**Fig. 5.**
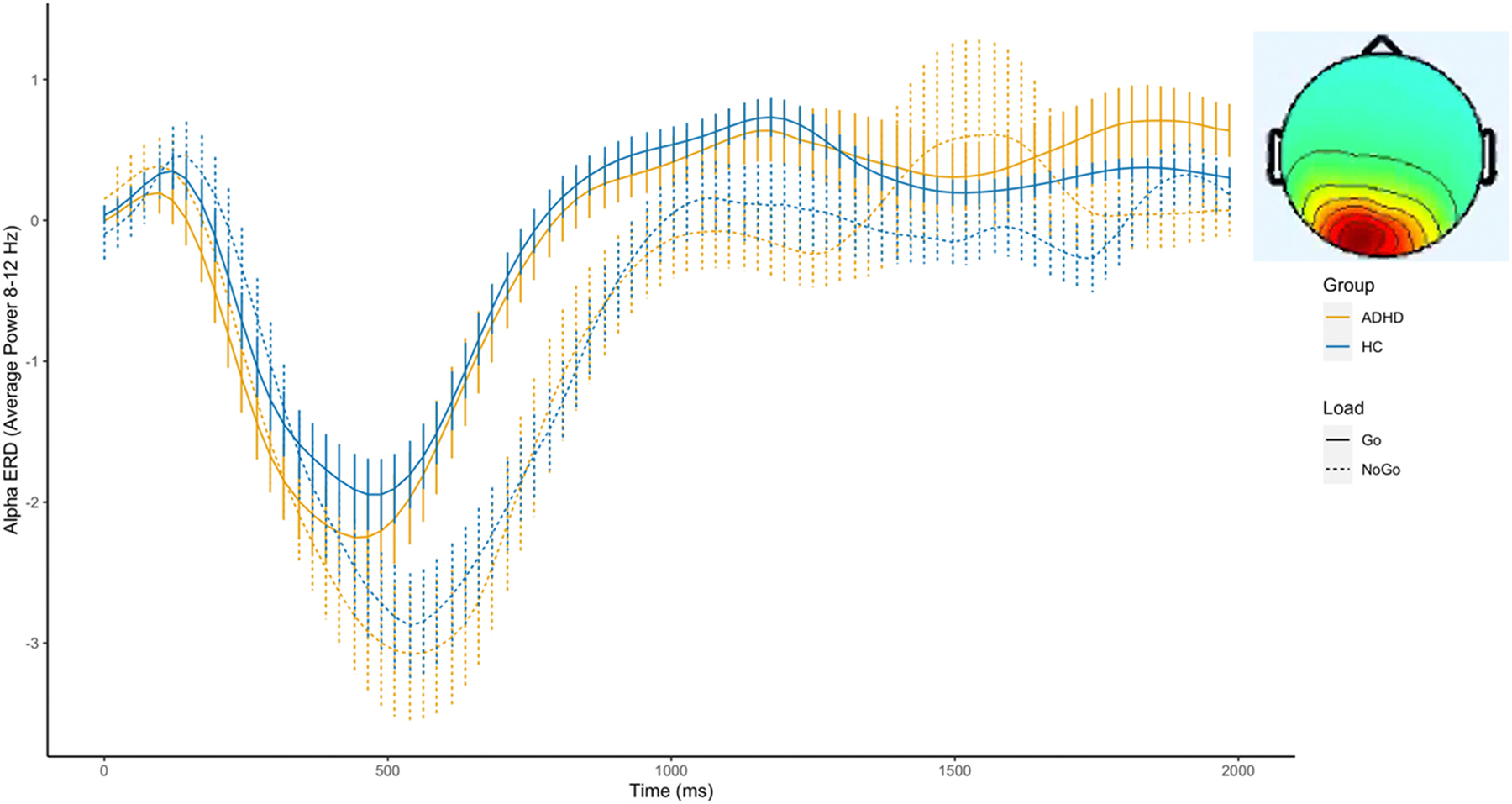
Alpha power in the Occipital cluster during the CPT task.

An additional goal of our analysis was to elucidate the temporal dynamics of alpha ERD by evaluating alpha power continuously over time, and thus, we visualize the averaged alpha ERD time course (as compared with traditional time-frequency visualizations). This approach offers a distinct advantage: it allows for a clearer visualization of variations in latencies and trajectories between conditions, such as the extended alpha latency observed in the NoGo compared to the Go condition (see Fig. 5). Moreover, this method of visualization further highlights the variability inherent in the alpha profile given significant fluctuations observed at different moments within a trial. For example, we found significant differences in alpha ERD magnitude between the encoding and probe phases of the SDRT task. The latter showed less sensitivity to load effects. Future studies will be needed to parse this heterogeneity and test whether there are underlying subgroups in alpha trajectories that may be linked to specific cognitive impairments or clinical characteristics.

### 4.5. Limitations and concluding comments

Several limitations should be noted. First, our analysis focused exclusively on alpha ERD considering its role in attentional processes and cognitive impairments. However, there may be contributions of other frequency bands that may account for potential compensatory mechanisms. Future research should extend beyond alpha band to investigate a broader spectrum of neural responses present in adult ADHD. Second, our control group consisted of individuals without a lifetime history of ADHD, however, they may still show other possible comorbidities. This group consisted of first-degree relatives of children with ADHD, so they may likely carry greater ADHD-related risks than individuals without any family history. Third, our speculations regarding potential developmental processes occurring between childhood and adulthood are based on cross-sectional data, and thus, this restricts our ability to draw causal inferences and observe developmental patterns.

## 5. Concluding comments

The neurophysiological signature of cognitive impairments present in adult ADHD remains poorly understood. Our results speak to the robustness of alpha as a marker of visual attention (i.e., significant associations with behavioral performance). Contrary to expectations, we observed no consistent occipital differences in alpha ERD between ADHD and control groups. However, we found group differences for centro-occipital alpha sources, suggesting a potential normalization of visuospatial processes with development in adults with a history of ADHD. Future research should consider longitudinal designs to elucidate developmental trajectories and understand the heterogeneity within the ADHD spectrum.

## Supplementary Material

SupplementaryMaterial

## Figures and Tables

**Table 1:** Demographic and clinical sample characteristics.

	ADHD group	Control group	*p*

N	85	105	
% female	21%	27%	
Age (*M*, *SD*)	44.31 (6.14)	44.07 (6.02)	
Inattention subscale (*M*, *SD*)	11.46 (4.87)	5.46 (3.61)	*
Hyperactivity impulsivity subscale (*M*, *SD*)	8.75 (4.49)	4.39 (3.08)	*
**Spatial Delayed Response Task (SDRT)**			
Averaged load 1&3 RT (ms)	1095.06 (198.73)	1036.45 (175.55)	*
Averaged load 5 & 7 RT (ms)	1276.78 (200.77)	1238.91 (221.82)	
Averaged Load 1 & 3 RT SD (ms)	314.87 (92.90)	280.49 (78.66)	*
Averaged Load 5 & 7 RT SD (ms)	371.97 (109.45)	364.36 (109.97)	
Averaged Load 1 & 3 Accuracy	0.92 (0.09)	0.94 (0.09)	
Averaged Load 5 & 7 Accuracy	0.83 (0.12)	0.84 (0.12)	
**Continuous Performance Task (CPT)**			
Block 1 correct RT (ms)	1200.61 (202.91)	1151.96 (212.70)	
Block 2 correct RT (ms)	1164.00 (197.32)	1094.45 (184.69)	*
Block 1 Accuracy	0.87 (0.10)	0.88 (0.09)	
Block 2 Accuracy	0.90 (0.08)	0.90 (0.09)	

*Note. M* = Mean, *SD* = Standard deviations. The asterisk indicates significant (*p* < 0.05) effects. The inattention and hyperactivity-impulsivity subscales belong to the ADHD rating scale measure. A chi-square test of independence was used to examine sex differences between groups. Two sample *t*-tests were used to examine group differences in age, symptom, and behavioral measures.

## Data Availability

The authors do not have permission to share data.
