# Supplementary material for: Alpha event-related decreases during encoding in adults with ADHD – An investigation of sustained attention and working memory processes
Source: Behav Brain Res. Author manuscript; Available in PMC 2024 Dec 31. (PMC11687179; doi:10.1016/j.bbr.2024.115003)
Supplement: SupplementaryMaterial [file NIHMS2041068-supplement-SupplementaryMaterial.docx]

**Supplementary Materials**

**Alpha Event-Related Decreases During Encoding in Adults with ADHD – An Investigation of Sustained Attention and Working Memory Processes**

[Supplemental Materials Section 1.1: SDRT Behavioral Measures 2](#_Toc165527023)

[Supplemental Materials Section 1.2: CPT Behavioral Measures 3](#_Toc165527024)

[Supplemental Materials Section 2.1: SDRT Alpha in Frontal Cluster 5](#_Toc165527025)

[Supplemental Materials Section 2.2: SDRT Alpha in Central Cluster 8](#_Toc165527026)

[Supplemental Materials Section 2.3: SDRT Alpha in Central-Occipital Cluster 11](#_Toc165527027)

[Supplemental Materials Section 2.4: SDRT Alpha in Occipital Cluster 14](#_Toc165527028)

[Supplemental Materials Section 3.1: Associations SDRT Central-Occipital Alpha – Behavior/Symptoms 17](#_Toc165527029)

[Supplemental Materials Section 3.2: Associations SDRT Occipital Alpha – Behavior/Symptoms 19](#_Toc165527030)

[Supplemental Materials Section 4.1: CPT Alpha in Occipital and Central-Occipital Cluster 21](#_Toc165527031)

[Supplemental Materials Section 4.2: Associations CPT Occipital Alpha – Behavior/Symptoms 25](#_Toc165527032)

[Supplemental Materials Section 4.3: Associations CPT Central-Occipital Alpha – Behavior/Symptoms 26](#_Toc165527033)

[Supplemental Materials Section 5: Exploratory Analysis of the P3 27](#_Toc165527034)

[Supplementary Material Section 6: Channel Results 32](#_Toc165527035)

# Supplemental Materials Section 1.1: SDRT Behavioral Measures

**Figure S1**

*Scree plot for principal component analysis of SDRT measures*


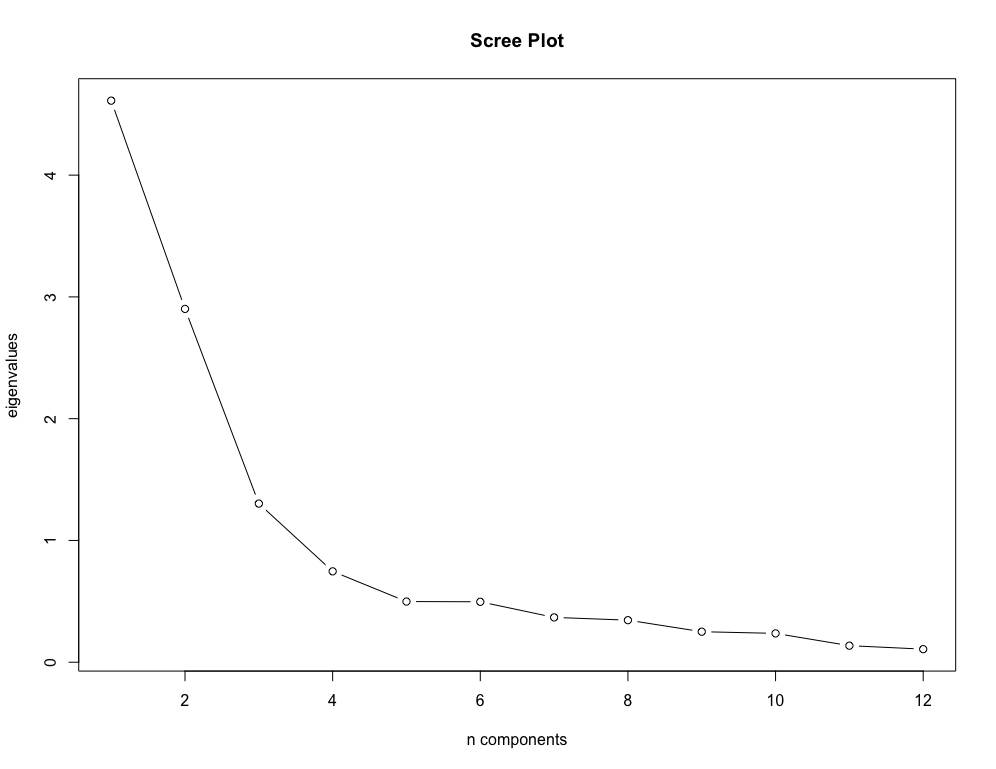


Based on the inspection of the scree plot and the Kaiser-Guttman rule (eigenvalues > 1), we selected three components. These components represent reaction-time, accuracy, and reaction time variability respectively.

# Supplemental Materials Section 1.2: CPT Behavioral Measures

**Figure S2**

*Scree plot for principal component analysis of CPT measures*


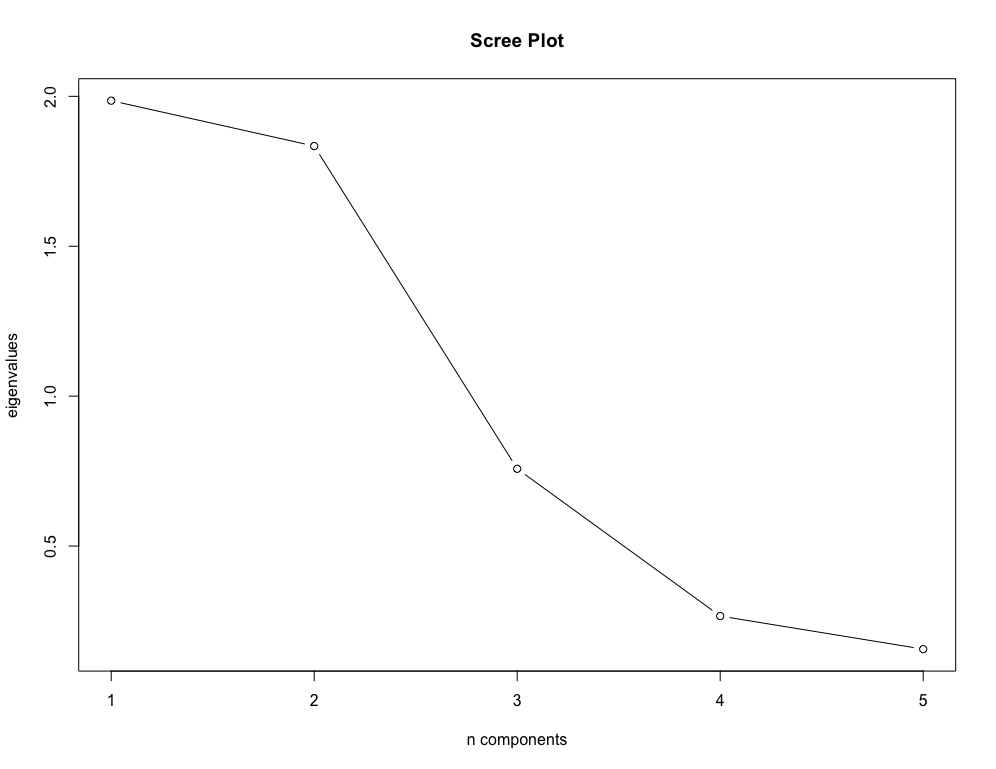


Based on the inspection of the scree plot and the Kaiser-Guttman rule (eigenvalues > 1), we selected two components. These two components represent reaction-time and accuracy respectively.

**Figure S3**

*Topographies for IC Clusters of Interest in the SDRT Task*


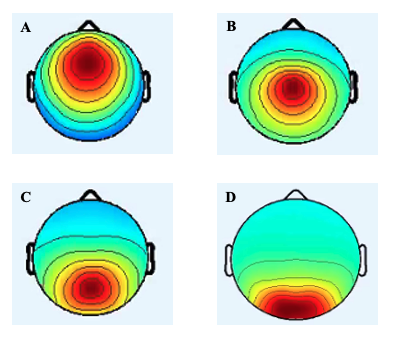


*Note.* Panel A (frontal cluster 9), panel B (central cluster 4), panel C (central-occipital cluster 14), panel D (occipital cluster 3).

# Supplemental Materials Section 2.1: SDRT Alpha in Frontal Cluster

**Figure S4**

*SDRT Frontal Cluster*


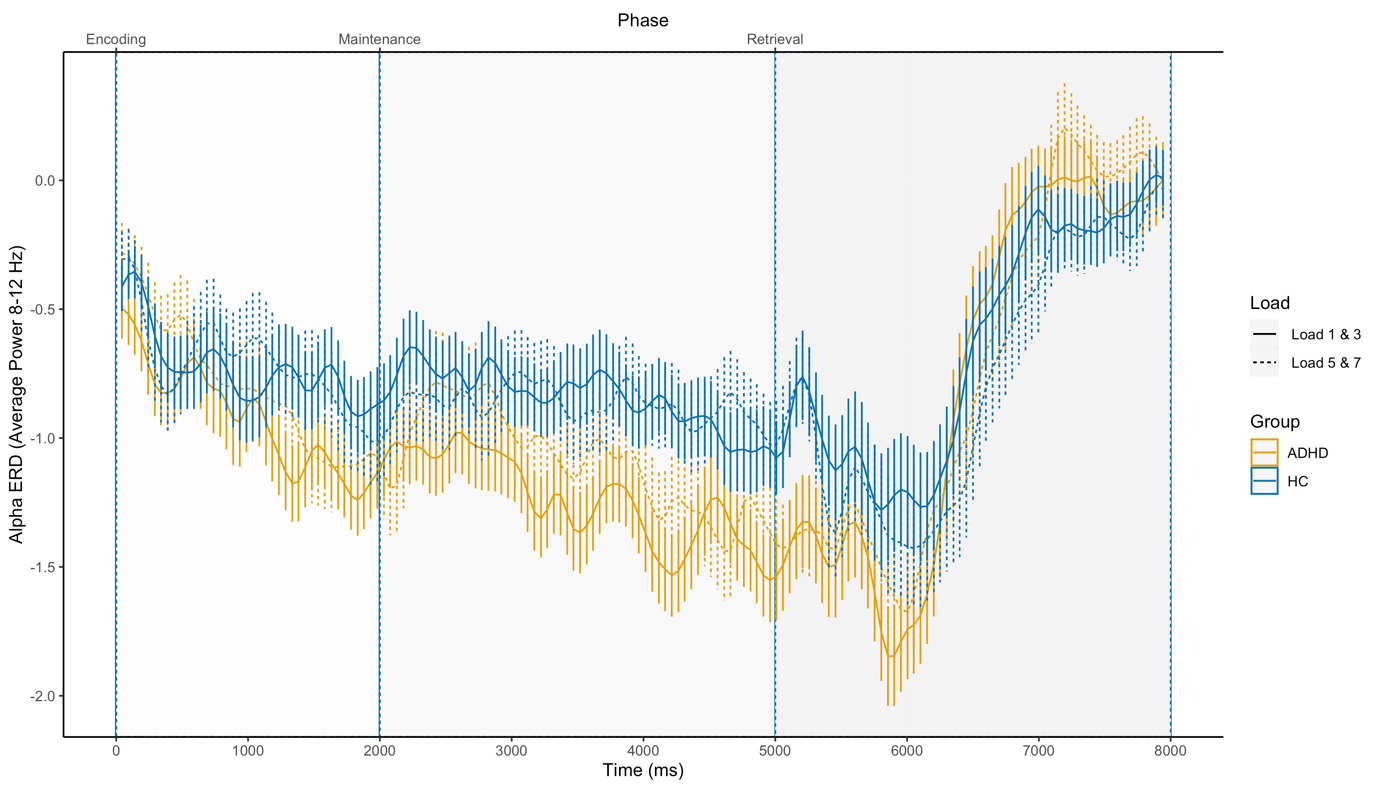


**Table S1**

*ANOVA results for 500ms window analysis of frontal cluster (SDRT)*

| Effect | test_window | F | p | p corrected |
| --- | --- | --- | --- | --- |
| Group | 0 - 500 | 0.001 | 0.975 | 0.975 |
| Load | 0 - 500 | 1.878 | 0.172 | 0.799 |
| Group:Load | 0 - 500 | 3.899 | 0.049 | 0.628 |
| Group | 500 - 1000 | 0.196 | 0.658 | 0.752 |
| Load | 500 – 1000 | 1.313 | 0.253 | 0.746 |
| Group:Load | 500 - 1000 | 0.035 | 0.853 | 0.853 |
| Group | 1000 - 1500 | 1.302 | 0.255 | 0.458 |
| Load | 1000 - 1500 | 1.172 | 0.28 | 0.746 |
| Group:Load | 1000 - 1500 | 0.085 | 0.77 | 0.853 |
| Group | 1500 - 2000 | 1.694 | 0.194 | 0.416 |
| Load | 1500 - 2000 | 0.023 | 0.878 | 0.894 |
| Group:Load | 1500 - 2000 | 0.579 | 0.447 | 0.67 |
| Group | 2000 - 2500 | 1.252 | 0.264 | 0.477 |
| Load | 2000 - 2500 | 0.084 | 0.773 | 0.894 |
| Group:Load | 2000 - 2500 | 1.034 | 0.31 | 0.628 |
| Group | 2500 - 3000 | 0.459 | 0.499 | 0.624 |
| Load | 2500 - 3000 | 0.201 | 0.654 | 0.894 |
| Group:Load | 2500 - 3000 | 2.053 | 0.153 | 0.628 |
| Group | 3000 - 3500 | 3.002 | 0.084 | 0.276 |
| Load | 3000 - 3500 | 1.023 | 0.313 | 0.799 |
| Group:Load | 3000 - 3500 | 0.759 | 0.384 | 0.64 |
| Group | 3500 - 4000 | 2.866 | 0.092 | 0.276 |
| Load | 3500 - 4000 | 0.518 | 0.472 | 0.885 |
| Group:Load | 3500 - 4000 | 1.434 | 0.232 | 0.628 |
| Group | 4000 - 4500 | 5.119 | 0.025 | 0.276 |
| Load | 4000 - 4500 | 0.797 | 0.373 | 0.799 |
| Group:Load | 4000 - 4500 | 1.179 | 0.279 | 0.628 |
| Group | 4500 - 5000 | 3.662 | 0.057 | 0.276 |
| Load | 4500 - 5000 | 0.878 | 0.35 | 0.799 |
| Group:Load | 4500 - 5000 | 0.143 | 0.705 | 0.813 |
| Group | 5000 - 5500 | 3.742 | 0.054 | 0.276 |
| Load | 5000 - 5500 | 0.018 | 0.894 | 0.894 |
| Group:Load | 5000 - 5500 | 0.31 | 0.578 | 0.723 |
| Group | 5500 - 6000 | 1.145 | 0.286 | 0.477 |
| Load | 5500 - 6000 | 0.048 | 0.826 | 0.894 |
| Group:Load | 5500 - 6000 | 2.312 | 0.13 | 0.628 |
| Group | 6000 - 6500 | 0.062 | 0.804 | 0.861 |
| Load | 6000 - 6500 | 2.032 | 0.155 | 0.799 |
| Group:Load | 6000 - 6500 | 1.332 | 0.25 | 0.628 |
| Group | 6500 - 7000 | 0.365 | 0.546 | 0.63 |
| Load | 6500 - 7000 | 8.397 | 0.004 | 0.06 |
| Group:Load | 6500 - 7000 | 0.042 | 0.838 | 0.898 |
| Group | 7000 - 7500 | 1.892 | 0.17 | 0.416 |
| Load | 7000 - 7500 | 0.195 | 0.659 | 0.894 |
| Group:Load | 7000 - 7500 | 0.438 | 0.509 | 0.694 |
| Group | 7500 - 8000 | 0.623 | 0.431 | 0.588 |
| Load | 7500 - 8000 | 0.095 | 0.759 | 0.894 |
| Group:Load | 7500 - 8000 | 0.932 | 0.335 | 0.628 |

# Supplemental Materials Section 2.2: SDRT Alpha in Central Cluster

Significant group differences were observed at the central alpha cluster, indicating stronger alpha ERD in the ADHD group compared to the control group during encoding. These differences were significant in the following time windows: 1500-2000ms F=4.39, p<0.05), 2000-2500ms (F=8.021, p<0.01), 3500-4000ms (F=4.127, p<0.05), 4000-4500ms (F=6.143, p<0.05), 4500-5000ms (F=4.57, p<0.05), and 5000-5500ms (F=4.503, p<0.05), although these differences did not remain significant after correcting for multiple comparisons. Regarding load effects, significant differences were noted in the 1500-2000ms window (F=15.85, p<0.01), with a stronger alpha ERD observed at higher loads.

**Figure S5**

*SDRT Central Cluster*


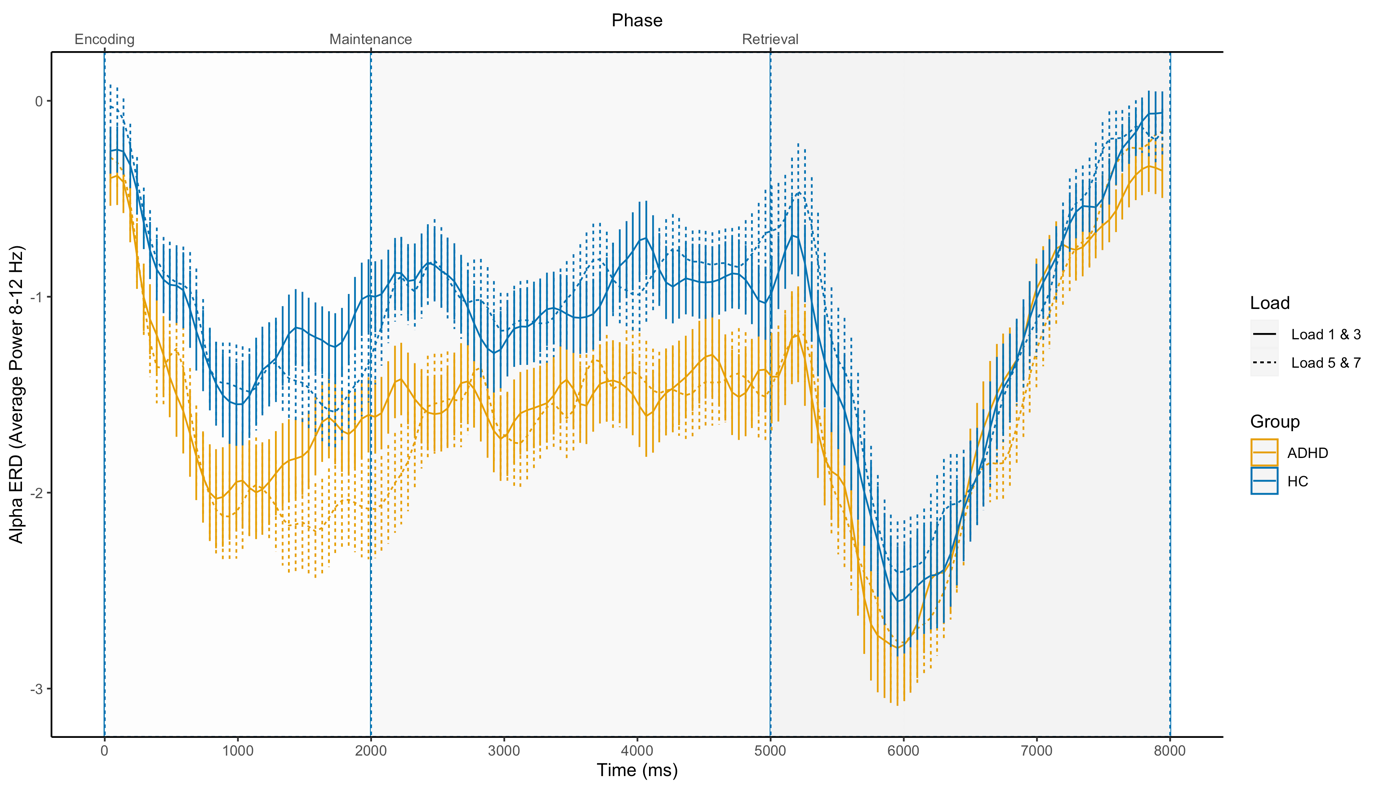


**Table S3**

*ANOVA results for 500ms window analysis of central cluster (SDRT)*

| Effect | Time window | F | p | p corrected |
| --- | --- | --- | --- | --- |
| Group | 0 - 500 | 3.596 | 0.059 | 0.11 |
| Load | 0 - 500 | 0.433 | 0.511 | 0.786 |
| Group:Load | 0 - 500 | 0.378 | 0.54 | 0.815 |
| Group | 500 - 1000 | 0.454 | 0.501 | 0.873 |
| Load | 500 - 1000 | 6.856 | 0.009 | 0.021 |
| Group:Load | 500 - 1000 | 0.156 | 0.693 | 0.875 |
| Group | 1000 - 1500 | 1.071 | 0.301 | 0.873 |
| Load | 1000 - 1500 | 43.127 | <0.01 | <0.01 |
| Group:Load | 1000 - 1500 | 0.608 | 0.436 | 0.875 |
| Group | 1500 - 2000 | 4.39 | 0.037 | 0.092 |
| Load | 1500 - 2000 | 15.85 | <0.01 | <0.01 |
| Group:Load | 1500 - 2000 | 0.279 | 0.598 | 0.815 |
| Group | 2000 - 2500 | 8.021 | 0.005 | 0.075 |
| Load | 2000 - 2500 | 3.626 | 0.058 | 0.415 |
| Group:Load | 2000 - 2500 | 1.393 | 0.239 | 0.815 |
| Group | 2500 - 3000 | 3.237 | 0.073 | 0.11 |
| Load | 2500 - 3000 | 0.416 | 0.519 | 0.786 |
| Group:Load | 2500 - 3000 | 0.023 | 0.879 | 0.965 |
| Group | 3000 - 3500 | 3.323 | 0.07 | 0.11 |
| Load | 3000 - 3500 | 0.272 | 0.602 | 0.786 |
| Group:Load | 3000 - 3500 | 0.355 | 0.552 | 0.815 |
| Group | 3500 - 4000 | 4.127 | 0.043 | 0.092 |
| Load | 3500 - 4000 | 0.399 | 0.528 | 0.786 |
| Group:Load | 3500 - 4000 | 0.003 | 0.953 | 0.965 |
| Group | 4000 - 4500 | 6.143 | 0.014 | 0.092 |
| Load | 4000 - 4500 | 0.02 | 0.887 | 0.887 |
| Group:Load | 4000 - 4500 | 0.002 | 0.965 | 0.965 |
| Group | 4500 - 5000 | 4.57 | 0.034 | 0.092 |
| Load | 4500 - 5000 | 0.234 | 0.629 | 0.786 |
| Group:Load | 4500 - 5000 | 0.763 | 0.383 | 0.815 |
| Group | 5000 - 5500 | 4.503 | 0.035 | 0.092 |
| Load | 5000 - 5500 | 1.713 | 0.192 | 0.72 |
| Group:Load | 5000 - 5500 | 0.899 | 0.344 | 0.815 |
| Group | 5500 - 6000 | 1.699 | 0.194 | 0.265 |
| Load | 5500 - 6000 | 1.065 | 0.303 | 0.786 |
| Group:Load | 5500 - 6000 | 0.477 | 0.491 | 0.815 |
| Group | 6000 - 6500 | 0.278 | 0.599 | 0.642 |
| Load | 6000 - 6500 | 0.132 | 0.716 | 0.826 |
| Group:Load | 6000 - 6500 | 1.568 | 0.212 | 0.815 |
| Group | 6500 - 7000 | 0.05 | 0.823 | 0.823 |
| Load | 6500 - 7000 | 3.041 | 0.083 | 0.415 |
| Group:Load | 6500 - 7000 | 1.601 | 0.207 | 0.815 |
| Group | 7000 - 7500 | 0.626 | 0.43 | 0.496 |
| Load | 7000 - 7500 | 0.028 | 0.867 | 0.887 |
| Group:Load | 7000 - 7500 | 0.341 | 0.56 | 0.815 |
| Group | 7500 - 8000 | 1.565 | 0.212 | 0.265 |
| Load | 7500 - 8000 | 0.992 | 0.32 | 0.786 |
| Group:Load | 7500 - 8000 | 0.793 | 0.374 | 0.815 |

# Supplemental Materials Section 2.3: SDRT Alpha in Central-Occipital Cluster

**Figure S6**

*Central-occipital cluster for the analysis of the SDRT task*


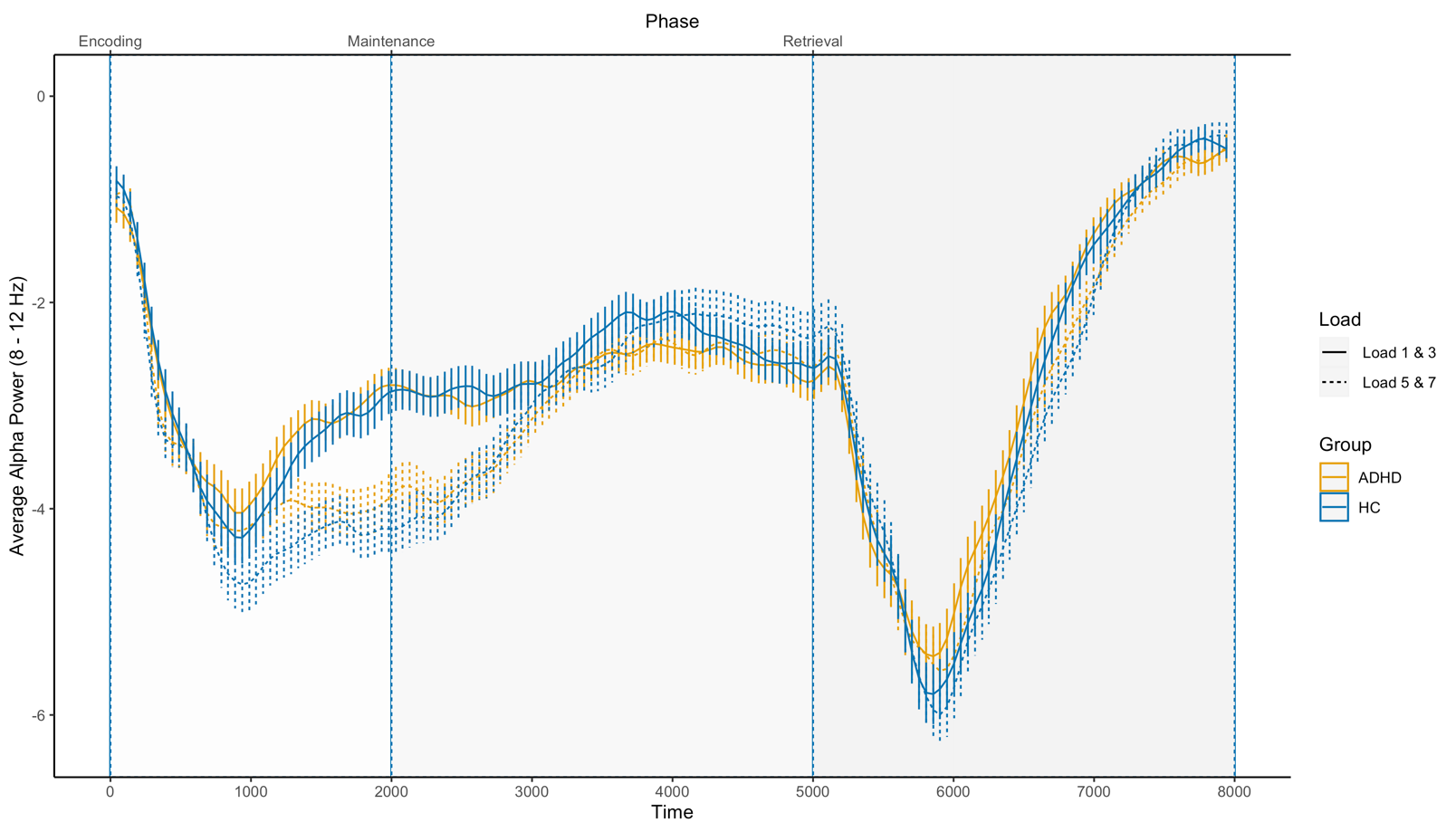


*Note.* The stimulus onset occurs at time = 0. Standard errors are shown in vertical bars.

**Table S5**

*ANOVA results for 500ms window analysis of central-occipital cluster (SDRT)*

| Effect | Time window | F | p | p_corrected |
| --- | --- | --- | --- | --- |
| Group | 0 - 500 | 0.002 | 0.964 | 0.964 |
| Load | 0 - 500 | 5.969 | 0.015 | 0.032 |
| Group:Load | 0 - 500 | 2.198 | 0.139 | 0.889 |
| Group | 500-1000 | 0.454 | 0.501 | 0.873 |
| Load | 500-1000 | 6.856 | 0.009 | 0.021 |
| Group:Load | 500-1000 | 0.156 | 0.693 | 0.875 |
| Group | 1000 - 1500 | 1.071 | 0.301 | 0.873 |
| Load | 1000 - 1500 | 43.127 | < 0.01 | < 0.01 |
| Group:Load | 1000 - 1500 | 0.608 | 0.436 | 0.875 |
| Group | 1500 - 2000 | 0.178 | 0.673 | 0.886 |
| Load | 1500 - 2000 | 95.255 | < 0.01 | < 0.01 |
| Group:Load | 1500 - 2000 | 0.352 | 0.553 | 0.889 |
| Group | 2000 - 2500 | 0.139 | 0.709 | 0.886 |
| Load | 2000 - 2500 | 100.292 | < 0.01 | < 0.01 |
| Group:Load | 2000 - 2500 | 1.154 | 0.283 | 0.889 |
| Group | 2500 - 3000 | 0.056 | 0.814 | 0.900 |
| Load | 2500 - 3000 | 25.619 | < 0.01 | < 0.01 |
| Group:Load | 2500 - 3000 | 0.001 | 0.972 | 0.972 |
| Group | 3000 - 3500 | 0.149 | 0.699 | 0.886 |
| Load | 3000 - 3500 | 1.187 | 0.277 | 0.378 |
| Group:Load | 3000 - 3500 | 0.138 | 0.711 | 0.889 |
| Group | 3500 - 4000 | 0.744 | 0.389 | 0.886 |
| Load | 3500 - 4000 | 0.7 | 0.403 | 0.504 |
| Group:Load | 3500 - 4000 | 0.871 | 0.351 | 0.889 |
| Group | 4000 - 4500 | 1.048 | 0.307 | 0.886 |
| Load | 4000 - 4500 | 0.317 | 0.574 | 0.615 |
| Group:Load | 4000 - 4500 | 0.177 | 0.675 | 0.889 |
| Group | 4500 - 5000 | 0.478 | 0.49 | 0.886 |
| Load | 4500 - 5000 | 2.479 | 0.116 | 0.193 |
| Group:Load | 4500 - 5000 | 0.323 | 0.57 | 0.889 |
| Group | 5000 - 5500 | 0.451 | 0.502 | 0.886 |
| Load | 5000 - 5500 | 2.647 | 0.105 | 0.193 |
| Group:Load | 5000 - 5500 | 0.015 | 0.904 | 0.969 |
| Group | 5500 - 6000 | 0.186 | 0.666 | 0.886 |
| Load | 5500 - 6000 | 0.327 | 0.568 | 0.615 |
| Group:Load | 5500 - 6000 | 0.049 | 0.825 | 0.952 |
| Group | 6000 - 6500 | 1.176 | 0.279 | 0.886 |
| Load | 6000 - 6500 | 7.31 | 0.007 | 0.018 |
| Group:Load | 6000 - 6500 | 0.15 | 0.698 | 0.889 |
| Group | 6500 - 7000 | 0.698 | 0.404 | 0.886 |
| Load | 6500 - 7000 | 35.363 | < 0.01 | < 0.01 |
| Group:Load | 6500 - 7000 | 0.245 | 0.621 | 0.889 |
| Group | 7000 - 7500 | 0.041 | 0.84 | 0.900 |
| Load | 7000 - 7500 | 2.285 | 0.131 | 0.196 |
| Group:Load | 7000 - 7500 | 1.054 | 0.305 | 0.889 |
| Group | 7500 - 8000 | 1.325 | 0.25 | 0.886 |
| Load | 7500 - 8000 | 0.011 | 0.915 | 0.915 |
| Group:Load | 7500 - 8000 | 0.244 | 0.622 | 0.889 |

# Supplemental Materials Section 2.4: SDRT Alpha in Occipital Cluster

**Figure S7**

*SDRT occipital cluster*

**
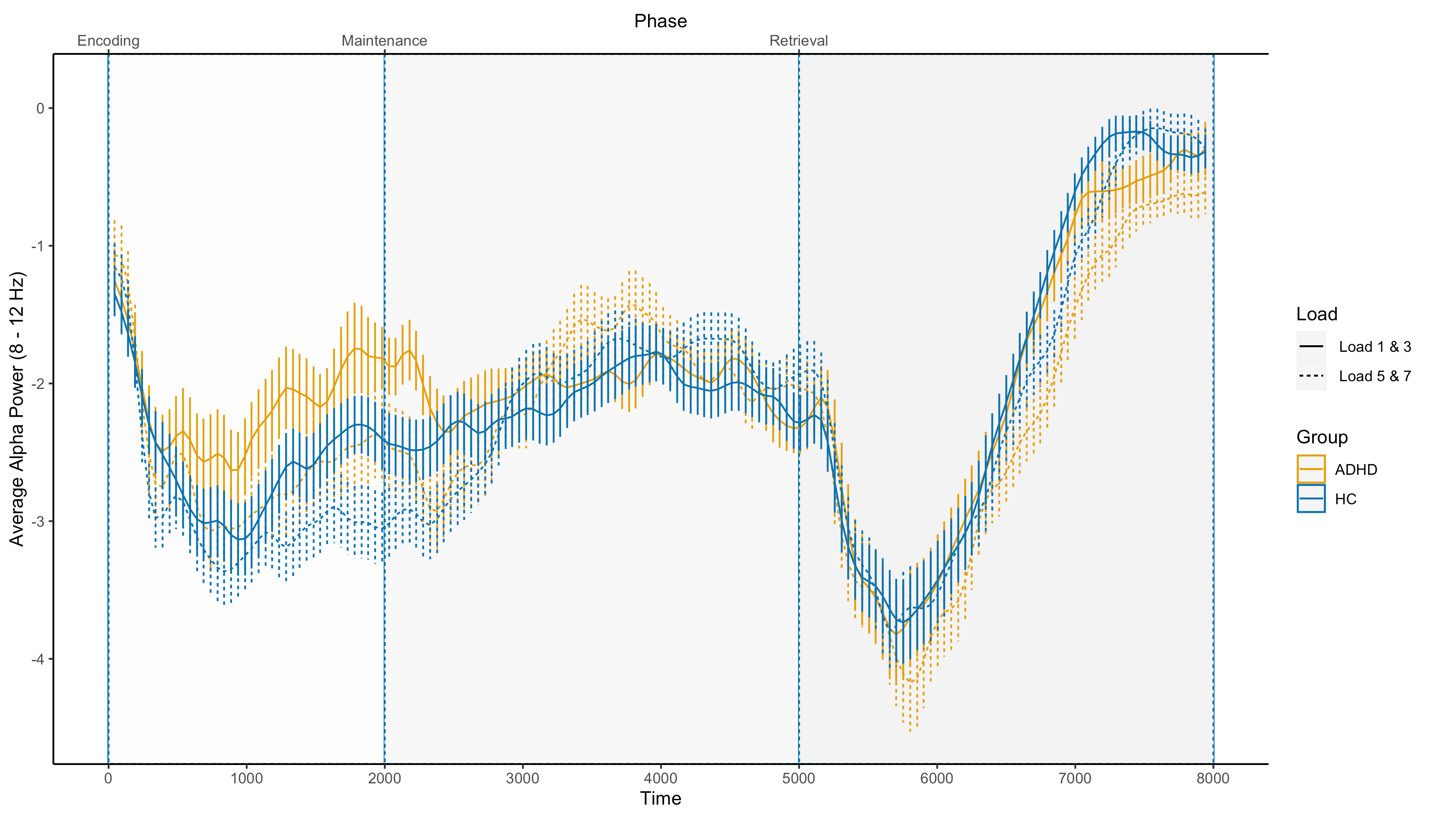
**

**Table S7**

*ANOVA results for 500ms window analysis of occipital cluster (SDRT)*

| Effect | Test window | F | p | p_corrected |
| --- | --- | --- | --- | --- |
| Group | 0 - 500 | 0.272 | 0.602 | 0.941 |
| Load | 0 - 500 | 1.091 | 0.297 | 0.343 |
| Group:Load | 0 - 500 | 0.403 | 0.526 | 0.717 |
| Group | 500-1000 | 1.094 | 0.297 | 0.792 |
| Load | 500-1000 | 7.559 | 0.006 | 0.016 |
| Group:Load | 500-1000 | 1.005 | 0.317 | 0.634 |
| Group | 1000 - 1500 | 1.559 | 0.213 | 0.792 |
| Load | 1000 - 1500 | 19.54 | < 0.01 | < 0.01 |
| Group:Load | 1000 - 1500 | 1.259 | 0.263 | 0.634 |
| Group | 1500 - 2000 | 2.006 | 0.158 | 0.79 |
| Load | 1500 - 2000 | 24.633 | < 0.01 | < 0.01 |
| Group:Load | 1500 - 2000 | 0.018 | 0.894 | 0.899 |
| Group | 2000 - 2500 | 1.109 | 0.293 | 0.879 |
| Load | 2000 - 2500 | 24.33 | < 0.01 | < 0.01 |
| Group:Load | 2000 - 2500 | 0.286 | 0.593 | 0.741 |
| Group | 2500 - 3000 | 0.068 | 0.795 | 0.941 |
| Load | 2500 - 3000 | 4.35 | 0.038 | 0.081 |
| Group:Load | 2500 - 3000 | 0.169 | 0.681 | 0.786 |
| Group | 3000 - 3500 | 0.269 | 0.604 | 0.941 |
| Load | 3000 - 3500 | 2.198 | 0.14 | 0.191 |
| Group:Load | 3000 - 3500 | 0.016 | 0.899 | 0.899 |
| Group | 3500 - 4000 | 0.065 | 0.8 | 0.941 |
| Load | 3500 - 4000 | 3.571 | 0.06 | 0.112 |
| Group:Load | 3500 - 4000 | 1.04 | 0.309 | 0.662 |
| Group | 4000 - 4500 | 0.003 | 0.956 | 0.956 |
| Load | 4000 - 4500 | 2.453 | 0.119 | 0.178 |
| Group:Load | 4000 - 4500 | 0.827 | 0.364 | 0.682 |
| Group | 4500 - 5000 | 0.04 | 0.841 | 0.941 |
| Load | 4500 - 5000 | 1.201 | 0.274 | 0.342 |
| Group:Load | 4500 - 5000 | 0.488 | 0.486 | 0.717 |
| Group | 5000 - 5500 | 0.024 | 0.878 | 0.941 |
| Load | 5000 - 5500 | 0.633 | 0.427 | 0.458 |
| Group:Load | 5000 - 5500 | 2.57 | 0.11 | 0.618 |
| Group | 5500 - 6000 | 0.136 | 0.713 | 0.941 |
| Load | 5500 - 6000 | 2.66 | 0.104 | 0.173 |
| Group:Load | 5500 - 6000 | 1.455 | 0.229 | 0.618 |
| Group | 6000 - 6500 | 0.086 | 0.769 | 0.941 |
| Load | 6000 - 6500 | 6.855 | 0.009 | 0.022 |
| Group:Load | 6000 - 6500 | 1.824 | 0.178 | 0.618 |
| Group | 6500 - 7000 | 0.811 | 0.369 | 0.922 |
| Load | 6500 - 7000 | 35.493 | < 0.01 | < 0.01 |
| Group:Load | 6500 - 7000 | 1.63 | 0.203 | 0.618 |
| Group | 7000 - 7500 | 4.599 | 0.033 | 0.495 |
| Load | 7000 - 7500 | 9.89 | 0.002 | 0.006 |
| Group:Load | 7000 - 7500 | 0.511 | 0.475 | 0.717 |
| Group | 7500 - 8000 | 2.846 | 0.093 | 0.698 |
| Load | 7500 - 8000 | 0.528 | 0.468 | 0.468 |
| Group:Load | 7500 - 8000 | 3.913 | 0.049 | 0.618 |

# Supplemental Materials Section 3.1: Associations SDRT Central-Occipital Alpha – Behavior/Symptoms

**Table S9**

*Regression results for associations between SDRT central-occipital alpha and symptom measures*

|  | Inattention Scale | | Hyperactivity Impulsivity | |
| --- | --- | --- | --- | --- |
| Alpha load 1&3,  0-500ms | -0.731*** |  | -0.370* |  |
|  | (0.237) |  | (0.207) |  |
|  |  |  |  |  |
| Alpha load 5&7,  0-500ms | | -0.168 |  | -0.117 |
|  |  | (0.239) |  | (0.208) |
|  |  |  |  |  |
| Constant | 6.882*** | 7.897*** | 5.476*** | 5.934*** |
|  | (0.765) | (0.814) | (0.680) | (0.725) |

**Table S10**

*Regression results for associations between SDRT central-occipital alpha and behavioral measures*

|  | PC1: RT | | PC2: Accuracy | | PC3: RT Variability | |
| --- | --- | --- | --- | --- | --- | --- |
| Alpha load 1&3,  0-500ms | -0.036 |  | 0.063 |  | 0.029 |  |
|  | (0.041) |  | (0.040) |  | (0.042) |  |
|  |  |  |  |  |  |  |
| Alpha load 5&7,  0-500ms | | -0.015 |  | 0.087** |  | 0.057 |
|  |  | (0.041) |  | (0.039) |  | (0.041) |
|  |  |  |  |  |  |  |
| Constant | 0.171 | 0.206 | 0.233* | 0.300** | 0.185 | 0.253* |
|  | (0.135) | (0.141) | (0.131) | (0.135) | (0.137) | (0.142) |
|  |  |  |  |  |  |  |

# Supplemental Materials Section 3.2: Associations SDRT Occipital Alpha – Behavior/Symptoms

**Table S11**

*Regression results for associations between SDRT occipital alpha and symptom measures*

|  | Inattention | Hyperactivity-impulsivity | | |
| --- | --- | --- | --- | --- |
| Alpha load 1&3,  0-500ms | 0.179 |  | 0.154 |  |
|  | (0.241) |  | (0.213) |  |
|  |  |  |  |  |
| Alpha load 5&7,  0-500ms | | 0.027 |  | 0.182 |
|  |  | (0.253) |  | (0.225) |
|  |  |  |  |  |
| Constant | 8.316*** | 7.987*** | 6.292*** | 6.339*** |
|  | (0.901) | (0.904) | (0.816) | (0.820) |

**Table S12**

*Regression results for associations between SDRT occipital alpha and behavioral measures*

|  | PC1: RT | | PC2: Accuracy | | PC3: RT Variability | |
| --- | --- | --- | --- | --- | --- | --- |
| Alpha load 1&3,  0-500ms | 0.047 |  | 0.041 |  | 0.026 |  |
|  | (0.043) |  | (0.042) |  | (0.042) |  |
|  |  |  |  |  |  |  |
| Alpha load 5&7,  0-500ms |  | 0.047 |  | 0.068 |  | 0.014 |
|  |  | (0.047) |  | (0.045) |  | (0.046) |
|  |  |  |  |  |  |  |
| Constant | 0.329** | 0.328** | 0.199 | 0.259 | 0.151 | 0.123 |
|  | (0.160) | (0.165) | (0.156) | (0.160) | (0.157) | (0.162) |

# Supplemental Materials Section 4.1: CPT Alpha in Occipital and Central-Occipital Cluster

**Figure S8**

*CPT Occipital Cluster*


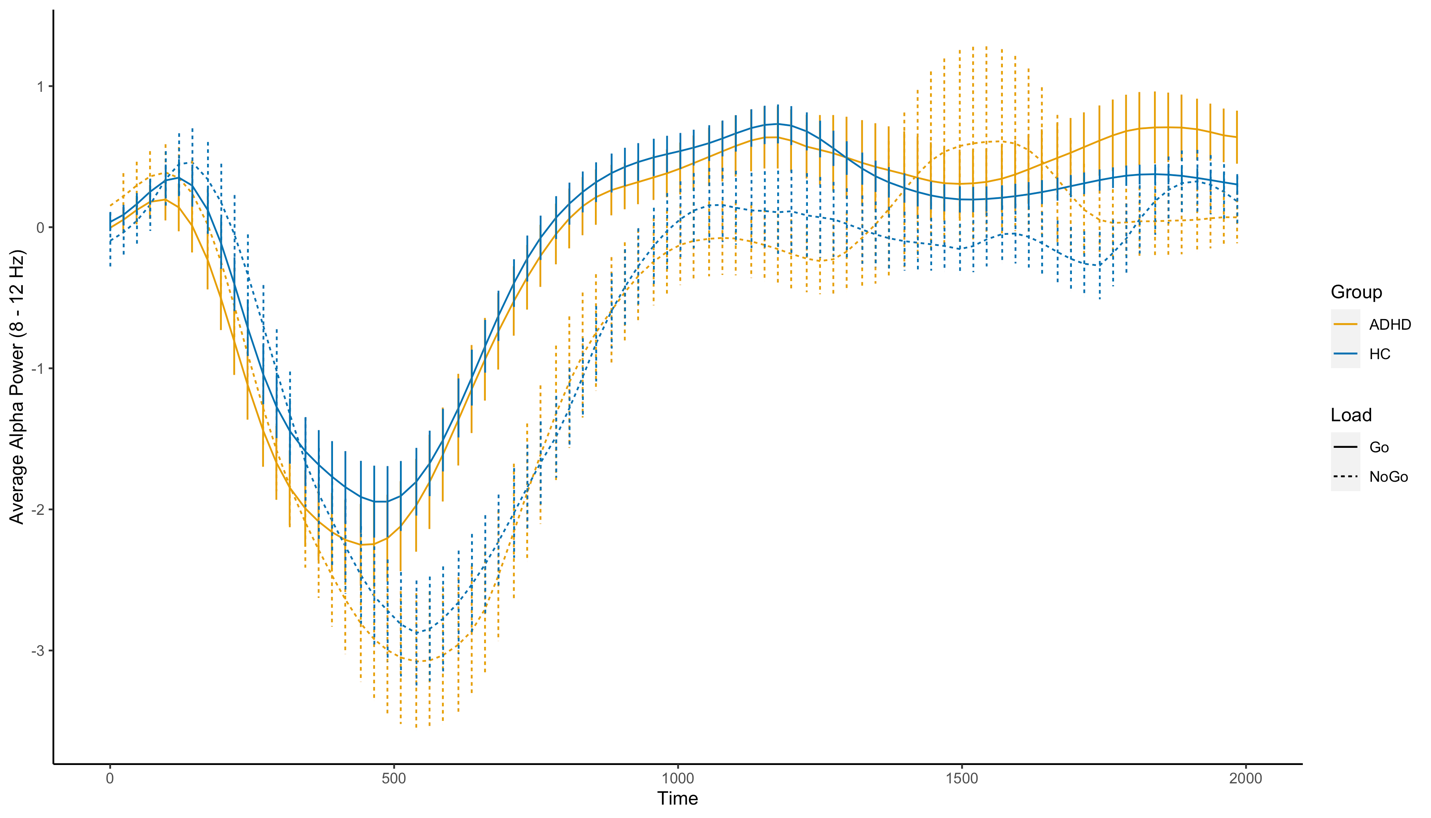


**Table S13**

*ANOVA results for 500ms window analysis of occipital cluster (CPT)*

| Effect | Test window | F | p |
| --- | --- | --- | --- |
| Group | 0 - 500 | 1.078 | 0.301 |
| Condition | 0 - 500 | 0.189 | 0.664 |
| Group:Condition | 0 - 500 | 0.013 | 0.91 |
| Group | 500 - 1000 | 0.108 | 0.743 |
| Condition | 500 – 1000 | 45.85 | < 0.01 |
| Group:Condition | 500 - 1000 | 0.004 | 0.947 |
| Group | 1000 - 1500 | 0.002 | 0.962 |
| Condition | 1000 - 1500 | 8.583 | 0.004 |
| Group:Condition | 1000 - 1500 | 0.001 | 0.97 |
| Group | 1500 - 2000 | 0.933 | 0.336 |
| Condition | 1500 - 2000 | 4.561 | 0.034 |
| Group:Condition | 1500 - 2000 | 0.016 | 0.900 |

**Figure S9**

*CPT Central-Occipital Cluster*


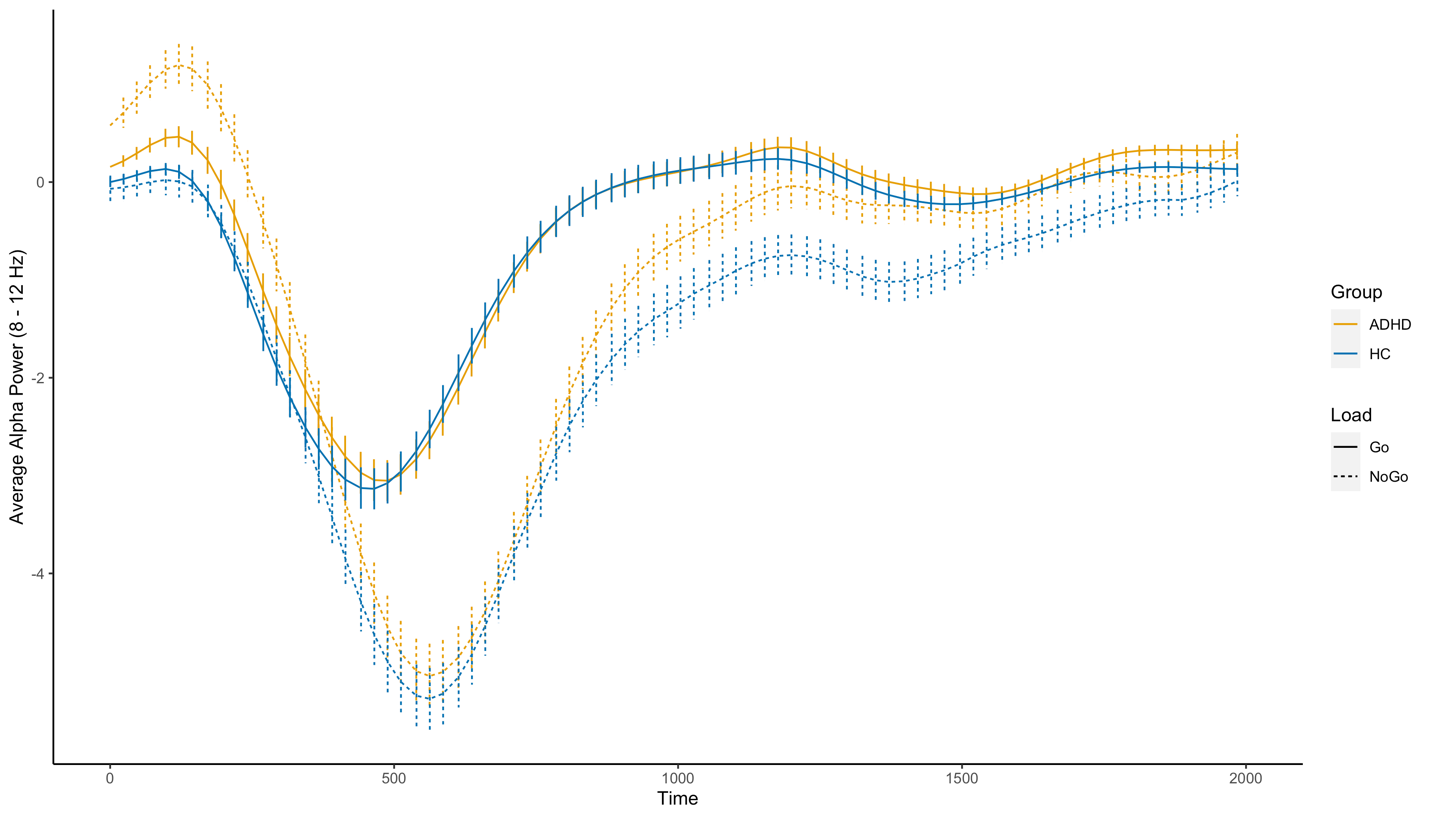


**Table S14**

*ANOVA results for 500ms window analysis of central-occipital cluster (CPT)*

| Effect | test window | F | p |
| --- | --- | --- | --- |
| Group | 0 - 500 | 6.894 | 0.009 |
| Condition | 0 - 500 | 0.092 | 0.762 |
| Group:Condition | 0 - 500 | 7.244 | 0.008 |
| Group | 500 - 1000 | 0.282 | 0.596 |
| Condition | 500 – 1000 | 238.795 | < 0.01 |
| Group:Condition | 500 – 1000 | 1.879 | 0.172 |
| Group | 1000 - 1500 | 4.574 | 0.033 |
| Condition | 1000 - 1500 | 27.917 | < 0.01 |
| Group:Condition | 1000 - 1500 | 5.372 | 0.021 |
| Group | 1500 - 2000 | 4.224 | 0.041 |
| Condition | 1500 - 2000 | 6.109 | 0.014 |
| Group:Condition | 1500 - 2000 | 0.933 | 0.335 |

# Supplemental Materials Section 4.2: Associations CPT Occipital Alpha – Behavior/Symptoms

**Table S15**

*Regression results for associations between CPT occipital alpha and symptom measures*

|  | Inattention | Hyperactivity-impulsivity | | |
| --- | --- | --- | --- | --- |
| Alpha go, 0-500ms | -0.275 |  | 0.183 |  |
|  | (0.498) |  | (0.416) |  |
|  |  |  |  |  |
| Alpha nogo, 0-500ms | | -0.121 |  | 0.202 |
|  |  | (0.405) |  | (0.332) |
|  |  |  |  |  |
| Constant | 8.196*** | 8.326*** | 6.418*** | 6.466*** |
|  | -1.059 | -1.046 | (0.882) | (0.874) |

**Table S16**

*Regression results for associations between CPT occipital alpha and behavioral measures*

|  | Inattention | Hyperactivity-impulsivity | | |
| --- | --- | --- | --- | --- |
| Alpha go, 0-500ms | -0.301 |  | -0.183 |  |
|  | (0.469) |  | (0.374) |  |
|  |  |  |  |  |
| Alpha nogo, 0-500ms | | -0.117 |  | -0.060 |
|  |  | (0.273) |  | (0.224) |
|  |  |  |  |  |
| Constant | 8.003*** | 8.186*** | 6.098*** | 6.207*** |
|  | (0.924) | (0.842) | (0.744) | (0.705) |

# Supplemental Materials Section 4.3: Associations CPT Central-Occipital Alpha – Behavior/Symptoms

**Table S17**

*Regression results for associations between CPT central-occipital alpha and symptom measures*

|  | Inattention | Hyperactivity-impulsivity | | |
| --- | --- | --- | --- | --- |
| Alpha go, 0-500ms | -0.301 |  | -0.183 |  |
|  | (0.469) |  | (0.374) |  |
|  |  |  |  |  |
| Alpha nogo, 0-500ms | | -0.117 |  | -0.060 |
|  |  | (0.273) |  | (0.224) |
|  |  |  |  |  |
| Constant | 8.003*** | 8.186*** | 6.098*** | 6.207*** |
|  | (0.924) | (0.842) | (0.744) | (0.705) |

**Table S18**

*Regression results for associations between CPT central-occipital alpha and behavioral measures*

|  | PC1_Acc_CPT | | PC2_RT_CPT | |
| --- | --- | --- | --- | --- |
| Alpha go, 0-500ms | -0.0003 |  | -0.064 |  |
|  | (0.064) |  | (0.069) |  |
|  |  |  |  |  |
| Alpha nogo, 0-500ms | | 0.027 |  | -0.038 |
|  |  | (0.041) |  | (0.044) |
|  |  |  |  |  |
| Constant | 0.319** | 0.358*** | 0.123 | 0.149 |
|  | (0.141) | (0.130) | (0.153) | (0.141) |

# Supplemental Materials Section 5: Exploratory Analysis of the P3

**ERP Results for SDRT**

**Figure S10**

*ERP in central-occipital SDRT cluster*


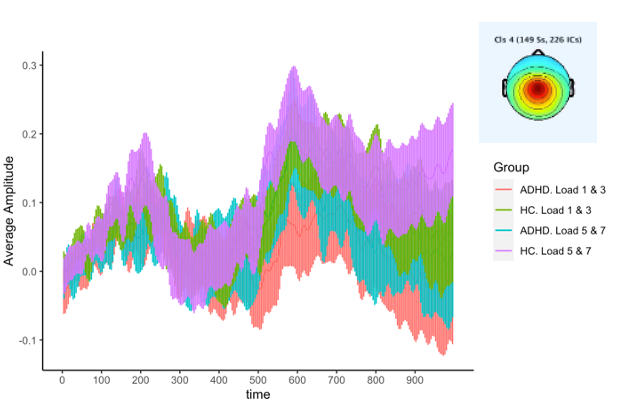


**Figure S11**

*ERP in occipital SDRT cluster*


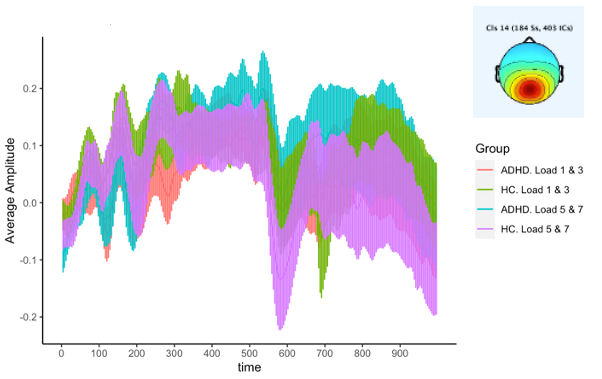


**ERP Results for CPT**

**Figure S12**

*ERP in central-occipital CPT cluster*


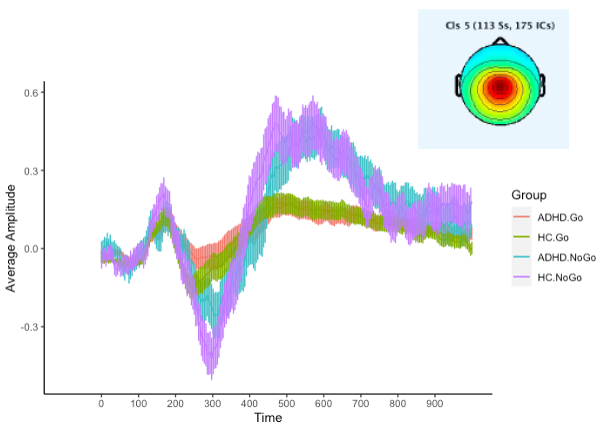


**Figure S13**

*ERP in occipital CPT cluster*


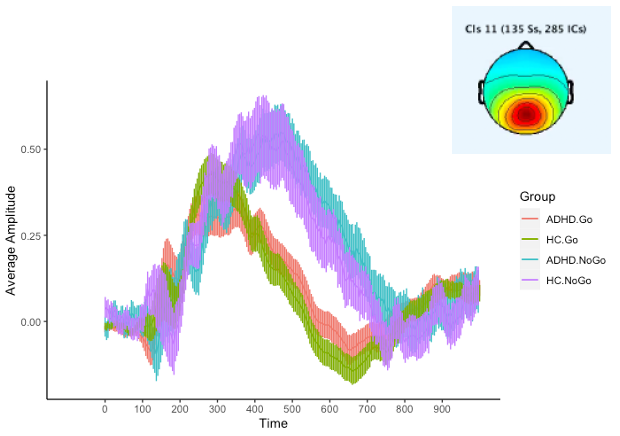


**Figure S14**

*Correlation between* occipital P3 amplitude and occipital encoding alpha in SDRT


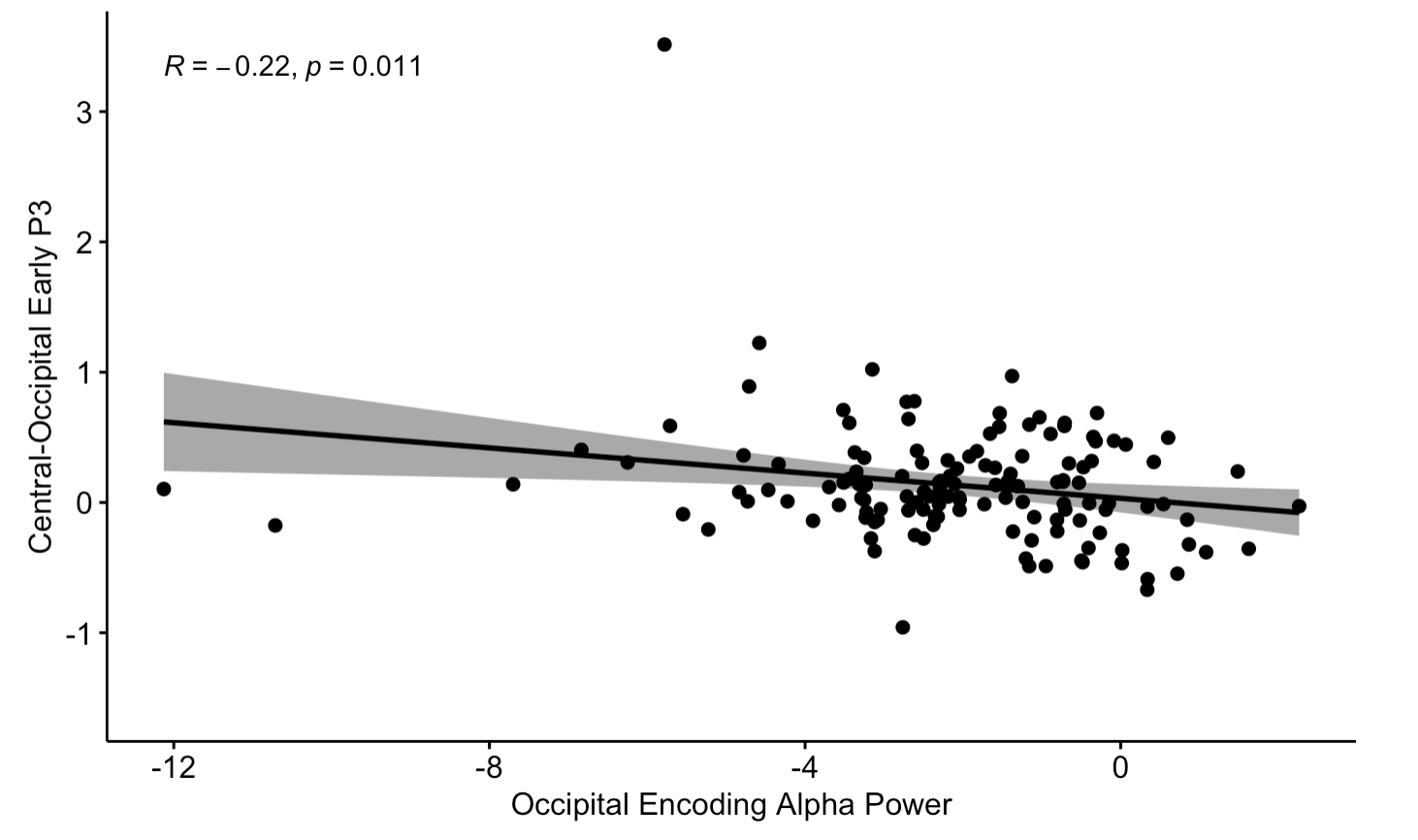


# Supplementary Material Section 6: Channel Results

**Figure S15**

*Time-frequency plot for Cz electrode in CPT*


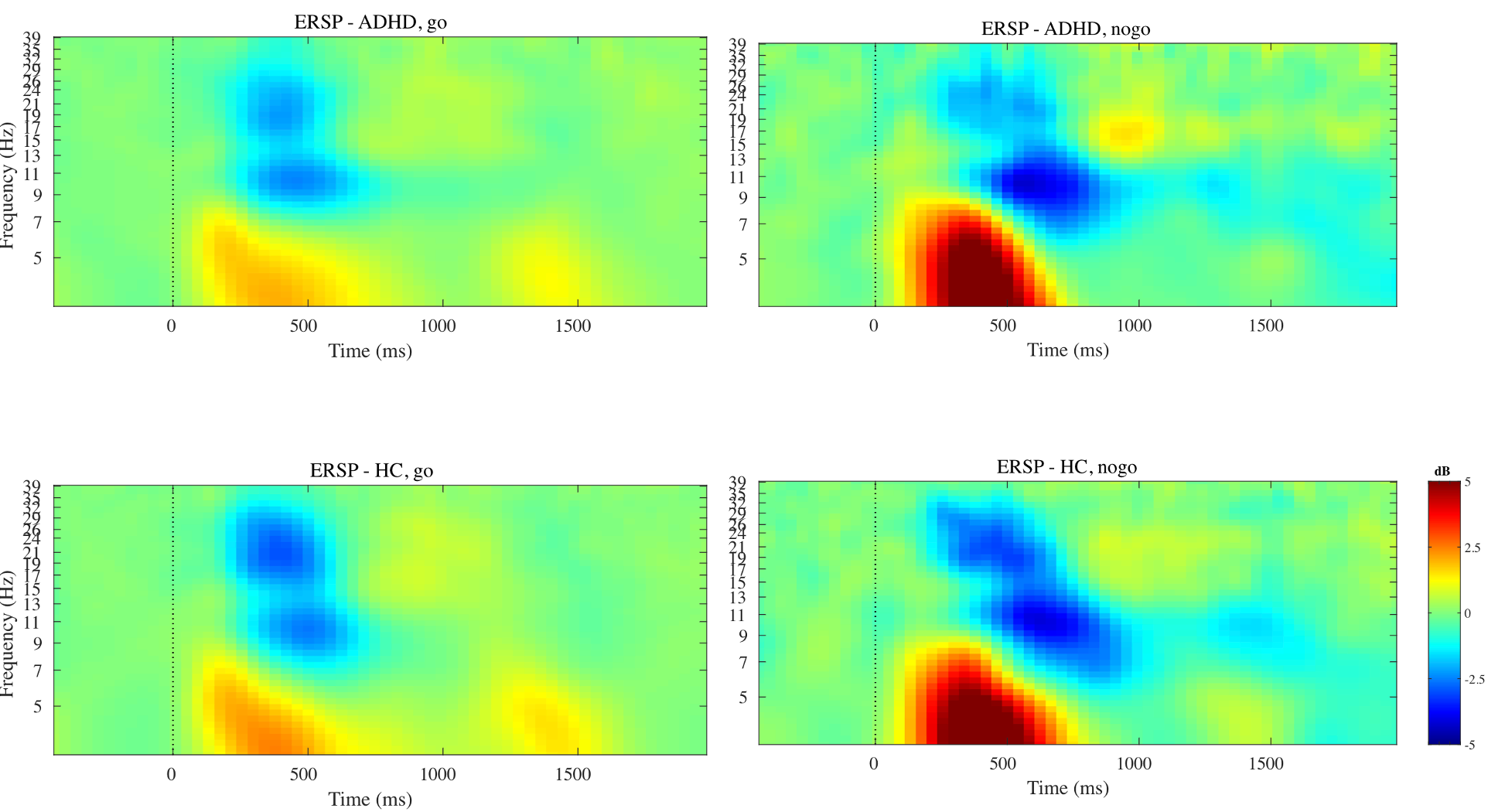


**Figure S16**

*Time-frequency plot for Fz electrode in CPT*
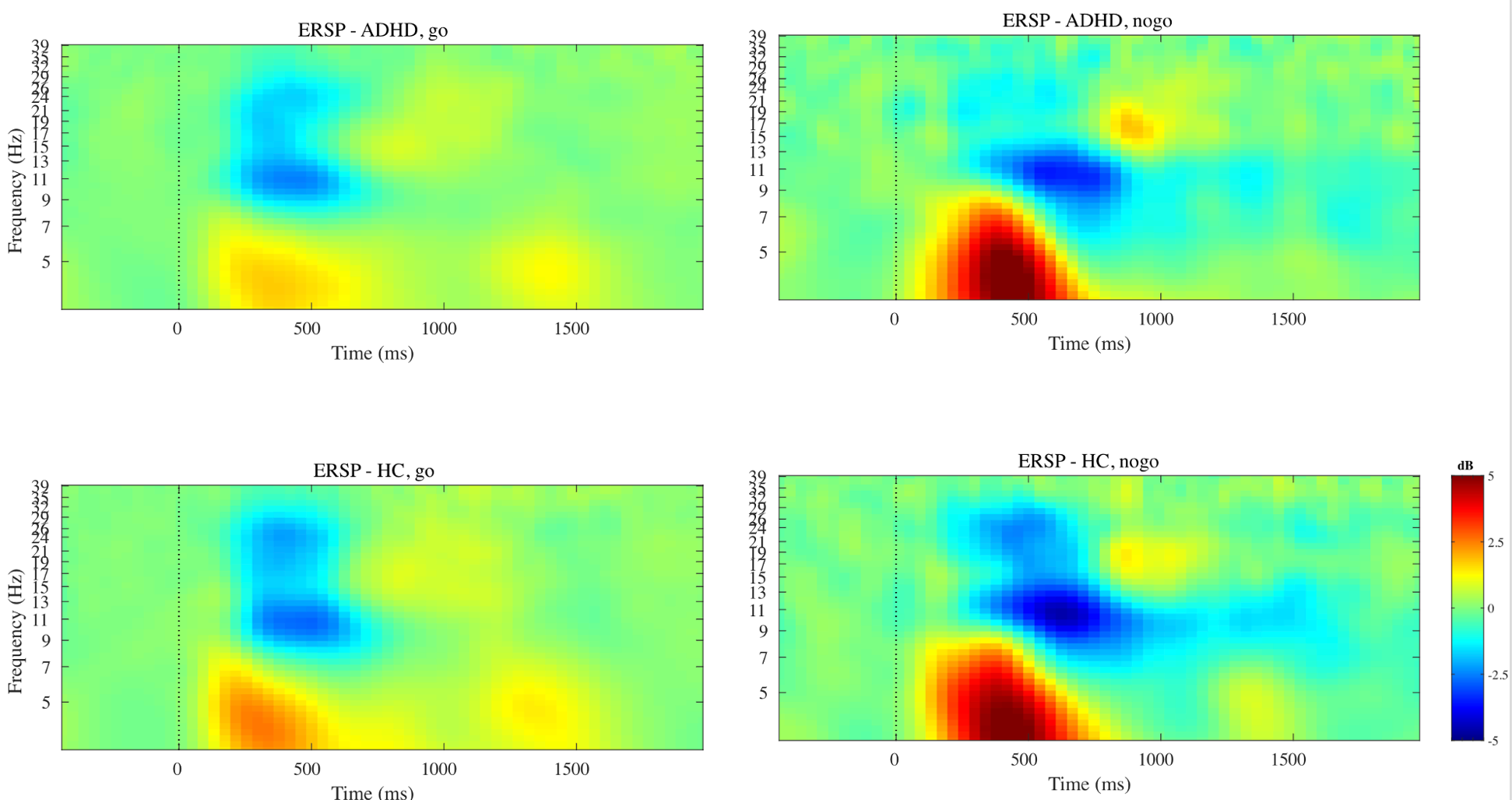


**Figure S17**

*Time-frequency plot for Oz electrode in CPT
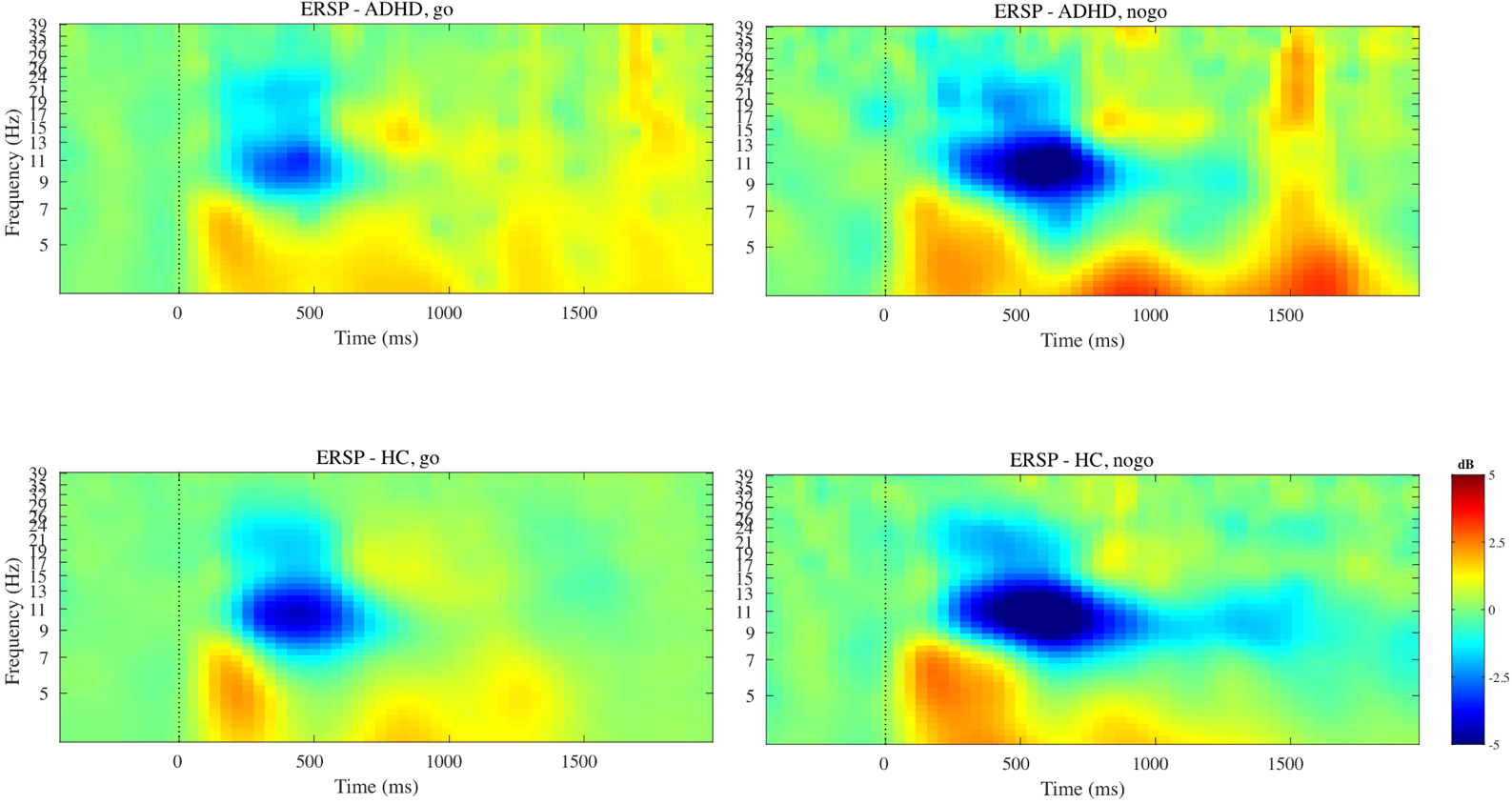
*

**Figure S18**

*Time-frequency plot for Pz electrode in CPT
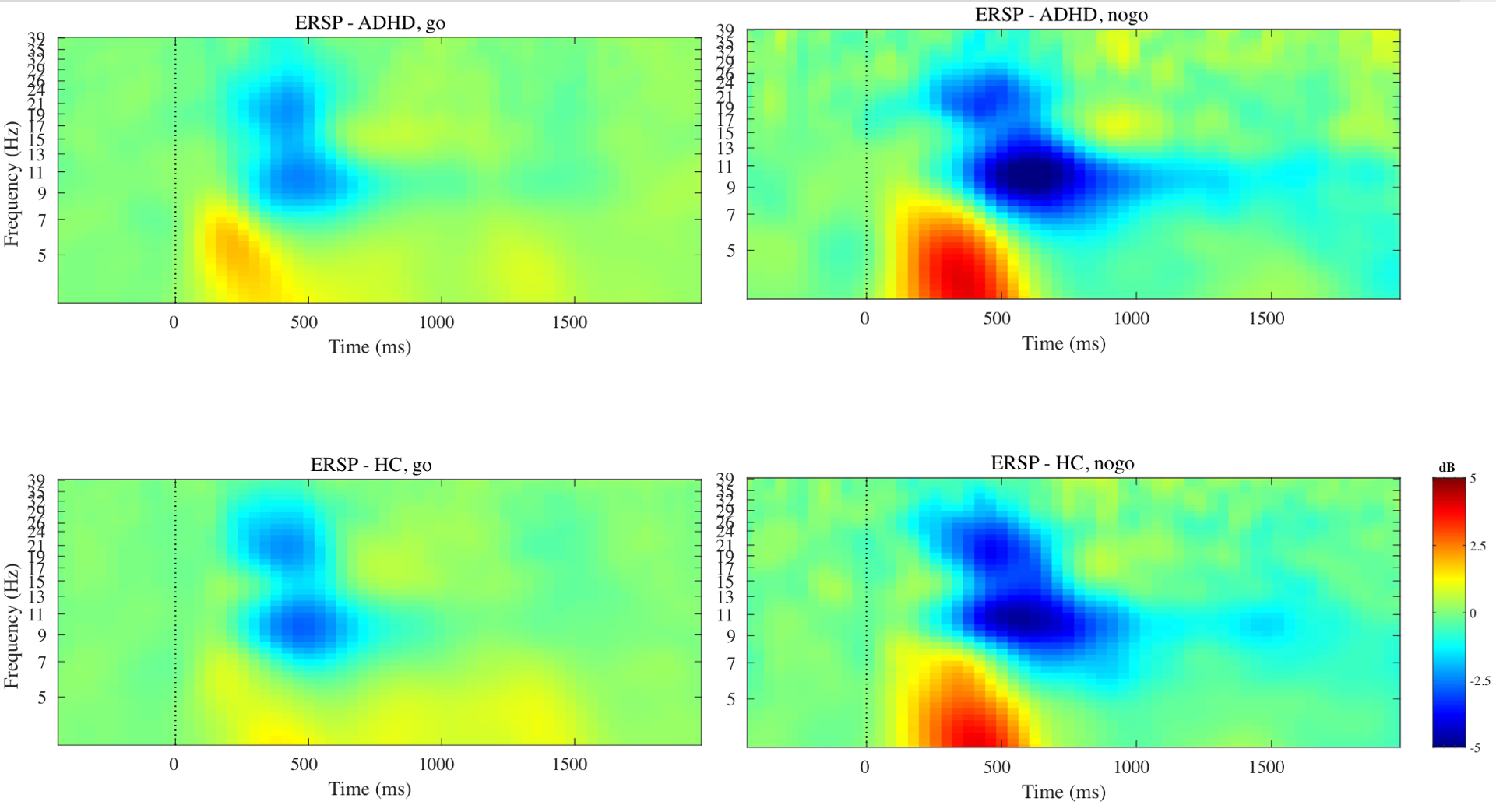
*

**Figure S19**

*Time-frequency plot for Cz electrode in SDRT*

*
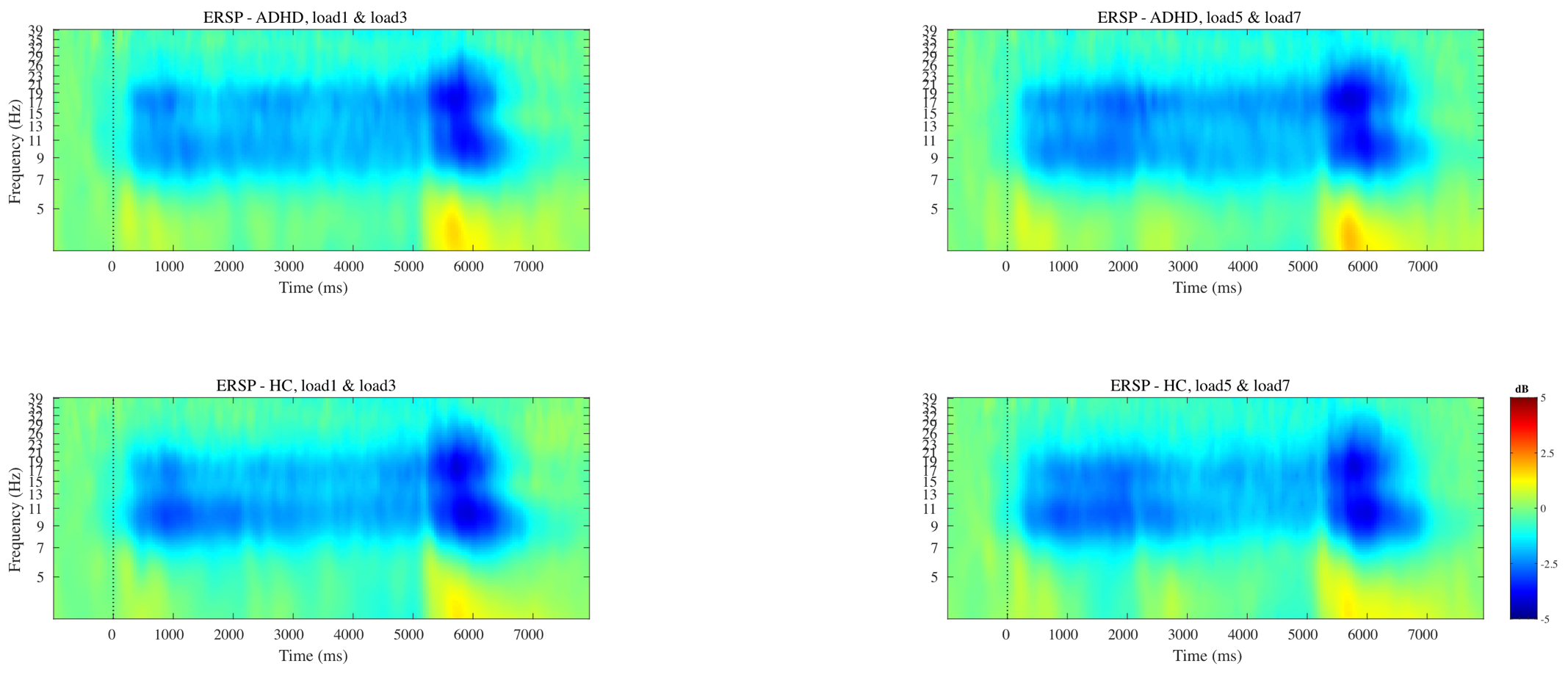
*

**Figure S20**

*Time-frequency plot for Fz electrode in SDRT*

*
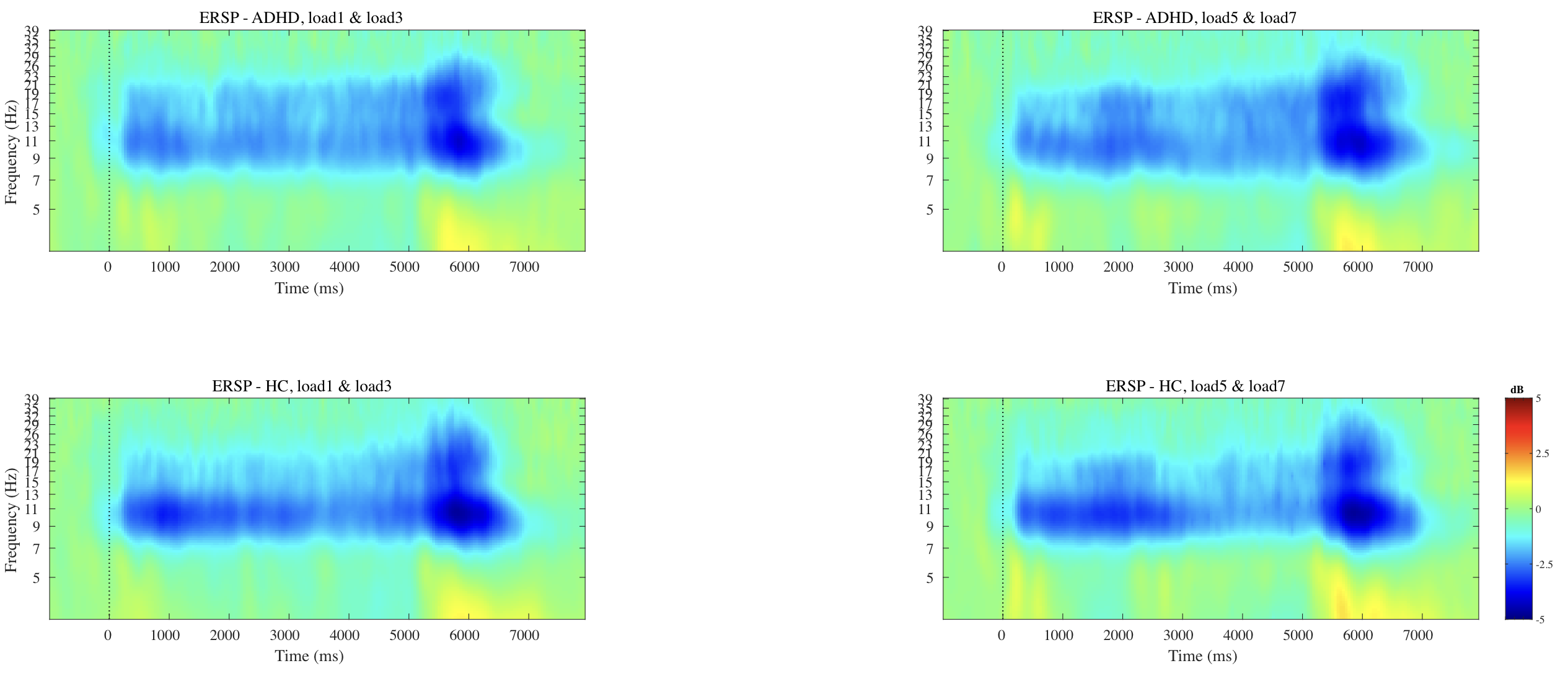
*

**Figure S21**

*Time-frequency plot for Oz electrode in SDRT*

*
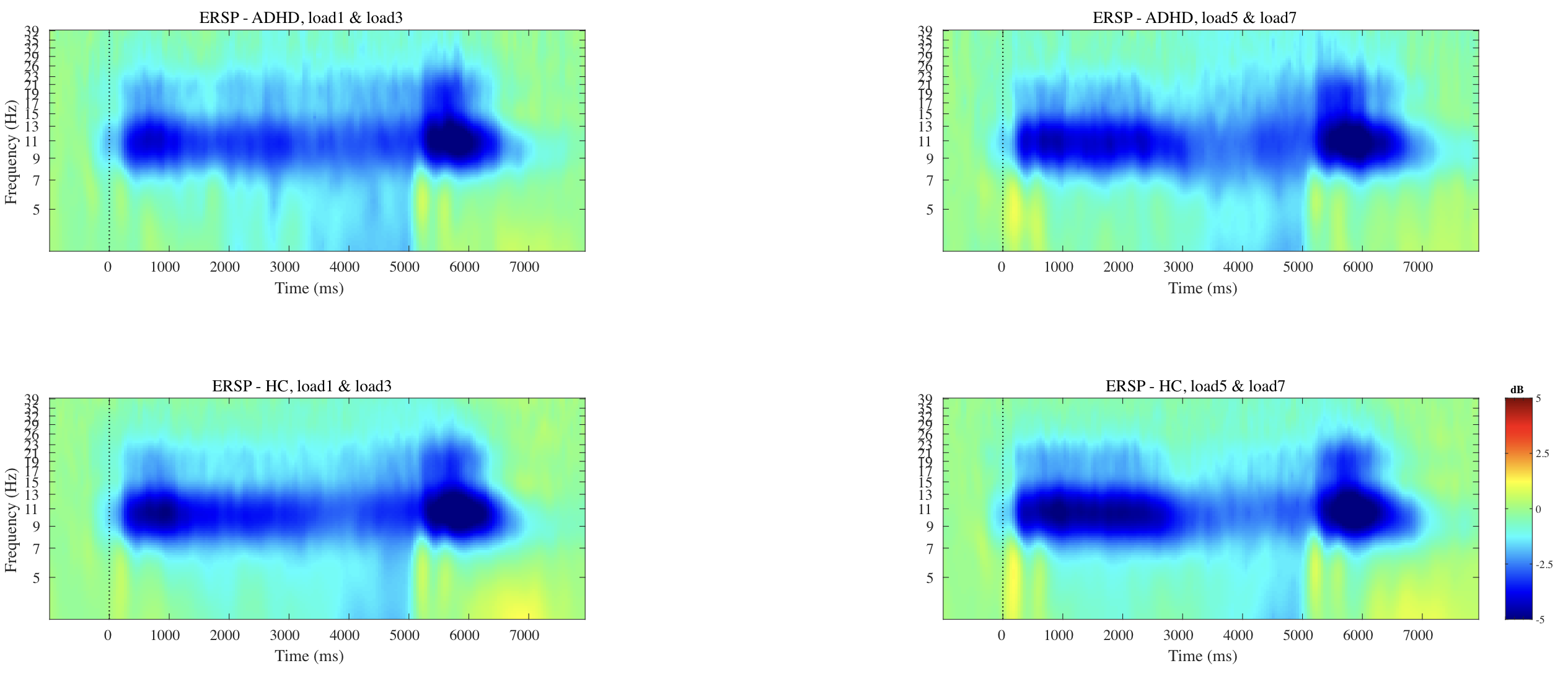
*

**Figure S22**

*Time-frequency plot for Pz electrode in SDRT*

*
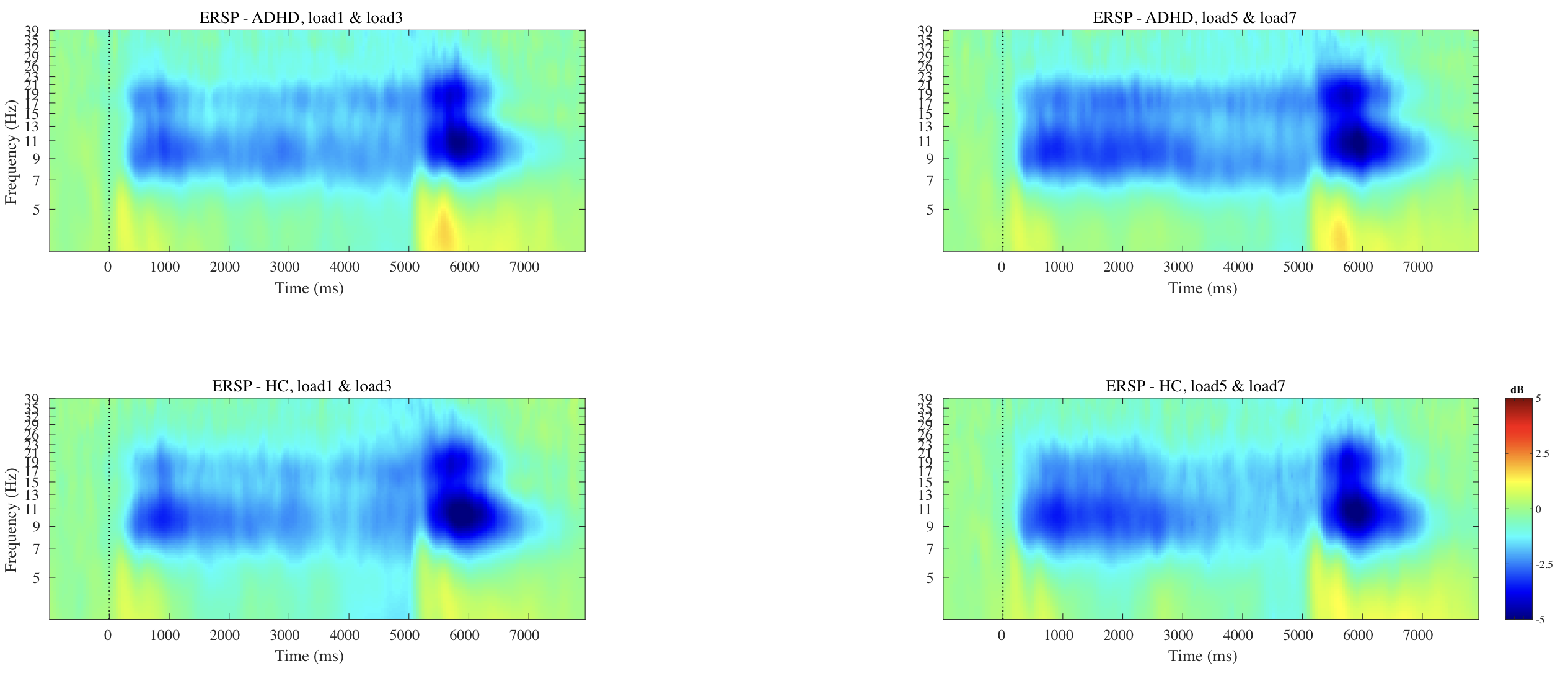
*
